# Bright insights into palladium-triggered local chemotherapy†
†Electronic supplementary information (ESI) available: Synthesis and characterization of materials, biological methods, *in vivo*/*ex vivo* protocols and Fig. S1–S13. See DOI: 10.1039/c8sc02291g


**DOI:** 10.1039/c8sc02291g

**Published:** 2018-07-17

**Authors:** Thomas L. Bray, Mark Salji, Alessandro Brombin, Ana M. Pérez-López, Belén Rubio-Ruiz, Laura C. A. Galbraith, E. Elizabeth Patton, Hing Y. Leung, Asier Unciti-Broceta

**Affiliations:** a Cancer Research UK Edinburgh Centre , Institute of Genetics and Molecular Medicine , University of Edinburgh , Crewe Road South , Edinburgh EH4 2XR , UK . Email: Asier.Unciti-Broceta@igmm.ed.ac.uk; b Institute of Cancer Sciences , University of Glasgow , Bearsden , Glasgow G61 1QH , UK . Email: Hing.Leung@glasgow.ac.uk; c CRUK Beatson Institute , Bearsden , Glasgow G61 1BD , UK; d MRC Human Genetics Unit , Institute of Genetics & Molecular Medicine , University of Edinburgh , Crewe Road South , Edinburgh EH4 2XR , UK

## Abstract

The incorporation of transition metal catalysts to the bioorthogonal toolbox has opened the possibility of producing supra-stoichiometric amounts of xenobiotics in living systems in a non-enzymatic fashion. For medical use, such metals could be embedded in implantable devices (*i.e.* heterogeneous catalyst) to “synthesize” drugs in desired locations (*e.g.* in a tumour) with high specificity and for extended periods of time, overcoming the useful life limitations of current local therapy modalities directed to specific organ sites (*e.g.* brachytherapy, controlled release systems). To translate this approach into a bona fide therapeutic option, it is essential to develop clinically-accessible implantation procedures and to understand and validate the activation process in relevant preclinical models. Herein we report the development of a novel Pd-activatable precursor of the red-fluorescent drug doxorubicin and Pd devices of optimized size and activity. Screening in state-of-the-art cancer models provided fundamental insights into the insertion protocols, safety and stability of the devices and into the prodrug distribution profile before and after activation.

## Abstract 2

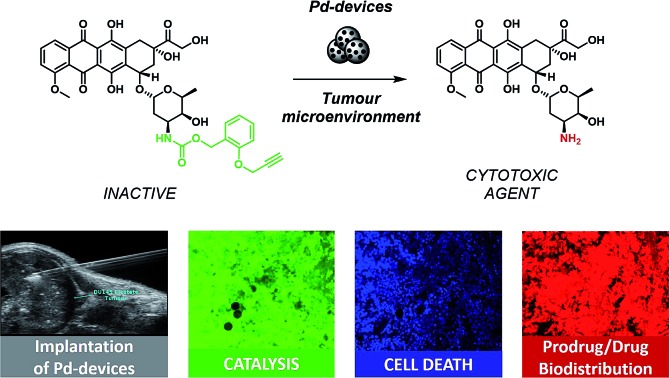
We report fundamental insights into the validity, reliability and clinical feasibility of using heterogeneous Pd catalysts as implantable devices to accurately activate chemotherapy within a tumour.

## Introduction

Advances in cancer diagnosis have led to major improvements in the early detection of clinically-localized prostate cancers.^1^ Because of the adverse effects of prostatectomy and external beam radiotherapy (impotence, incontinence, *etc.*), in most patients this low-to-moderate risk form of prostate cancer has been traditionally managed through active surveillance programs, which has resulted in an increment in the risk of dying from prostate malignancies.^2^,^3^ Focal therapies were introduced to facilitate localized tumour treatment with preservation of healthy prostate tissue, thus reducing the morbidity associated with whole-gland treatments without jeopardizing cancer control.^1^,^3^ Although current technologies mediating local treatment of disease (*e.g.* brachytherapy, external-beam focal radiotherapy, drug-eluting devices) can successfully improve a patient's quality of life, they have important limitations^4^ that either restrict their widespread clinical use (high cost of brachytherapy and external-beam focal radiotherapy) or the duration of their therapeutic effect (radioisotope's short half-life and limited drug cargo capacity of brachytherapy and controlled-release implants, respectively).

Our capacity to visualize and modulate physiological and pathological processes in the biological milieu has expanded enormously in the last 15 years through the development of a rich diversity of bioorthogonal tools and processes.^5^–^9^ Among them, the use of abiotic transition metals (ruthenium,^10^–^14^ palladium,^15^–^21^ copper,^22^–^24^ gold^25^–^27^) has emerged to facilitate the catalytic modification or manufacture of biomolecules and xenobiotics in living systems, contributing to the advent of a new prodrug modality that is not activated by a metabolic event but through a bioorthogonal bond cleavage reaction.^9^ Exploration of this novel prodrug paradigm in combination with heterogeneous Pd catalysis has stimulated the development of masking strategies that efficiently suppress the bioactivity of different classes of therapeutic agents while enabling selective drug activation – *via* Pd-mediated *N*- or *O*-dealkylation – both *in vitro* and *in vivo*.^20^,^28^–^31^ It is proposed that, by surgical implantation of Pd-containing inserts in a tumour followed by systemic administration of Pd-activatable therapeutic precursors, bioorthogonal drug release could take place exclusively in the disease site and minimise adverse effects in distant organs and tissues. Since the triggering mechanism is a catalytic process, multiple doses of one or more prodrugs could be intratumourally activated by the same implant to extend and/or customize the therapeutic intervention, thereby facilitating an adaptive form of local chemotherapy that can respond to the severity and progression of the disease. Although studies in mammal models supporting the effectiveness of such an approach to treat tumours locally have not yet been reported, recent studies with Pd-based nanostructures have shown their capacity to generate tumour-controlling levels of drugs in xenografts in mice.^32^,^33^ However, the development of cancer-targeting nanotechnologies remain a significant challenge by itself.^34^

From a translational perspective, the use of activating devices that can be surgically implanted in or near the tumour using well-established clinical procedures^3^,^35^ would ensure that the release of cytotoxic agents occurs exclusively at the disease site. Essential questions such as appropriate implantation protocols, stability and safety of the catalyst *in vivo* and optimal biodistribution of the prodrug before and after activation need to be first addressed to translate this novel therapeutic modality into a medically-viable alternative to brachytherapy and drug eluting devices. In our path towards this goal, herein we report the development of Pd devices of optimal size for ultrasound-guided intratumoural insertion and Pd-activatable prodrugs of doxorubicin: a strongly fluorescent cytotoxic drug that allowed us to monitor biodistribution in prostate cancer explants. Screening in state-of-the-art cancer models provided fundamental insights into the safety and catalytic properties of the activating device, and into the prodrug activation process.

## Results and discussion

### Design and synthesis of bioorthogonal prodrugs of doxorubicin

**Scheme 1:**
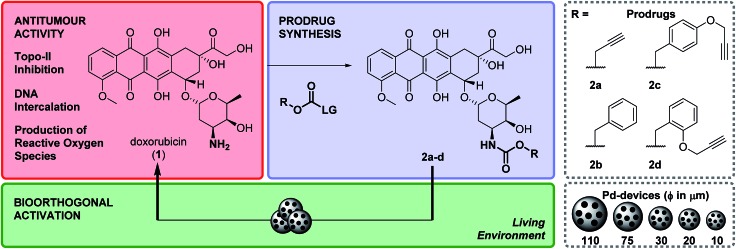
Overview of the strategy and research work. Prodrugs **2a–d** were prepared by reacting **1** with their corresponding 4-nitrophenyl (LG) carbonate. Pd-devices of a range of sizes (from 110 to 10 μm in diameter) were developed to investigate prodrug activation in biocompatible conditions, in cell culture and in prostate cancer explants.

Doxorubicin, **1**, is an anthracycline anticancer antibiotic used alone or in combination in the treatment of a wide range of hematological and solid malignancies.^36^ After entering cells by diffusion, this potent cytotoxic drug engages with various cytoplasmic and nuclear targets to synergistically disrupt cellular function in cancer cells and induce apoptosis (Scheme 1).^37^**1** has a multitude of moderate to severe side effects that negatively impact on its therapeutic window, limiting its maximum tolerated dose and frequency of administration (typically administered as a single intravenous infusion in days 1 and 2 of 21 day cycles). The most serious adverse effect of **1** is cardiotoxicity, typically observed by an early onset of cardiomyopathy that may progress to myocardial infarction.^38^ As a result, much of the research pertaining to **1** and their anthracycline analogues focuses on suppressing the cardiotoxic effect of this class of cytotoxic drugs. A variety of prodrug strategies are currently under investigation to improve tumour targetability^39^–^43^ and have shown that blockade of the primary amino group of the daunosamine moiety of **1** significantly reduces the bioactivity of the resulting derivative. Based on this evidence, the masking of the NH_2_ group as a carbamate group was investigated to develop novel biochemically-stable, Pd-sensitive prodrugs of **1** (Scheme 1).

Four carbamate derivatives of **1** were prepared as shown in Scheme 1 (see full experimental protocols in the ESI†). As the propargyloxycarbonyl (Poc) has been previously shown to be relatively resistant to metabolic activation and sensitive to Pd catalysis, derivative **2a** was developed as a positive control, whereas the Cbz-protected analogue **2b** (which is expected to be even more biochemically stable than Poc but unreactive to Pd catalysis under physiological conditions^28^) was used as a negative control. With the aim of increasing steric hindrance to further protect the carbamate bond from unspecific enzymatic cleavage, novel compounds containing a propargyloxybenzyl carbonyl (PBC) group were developed to enhance the metabolic stability of the derivatives whilst providing sensitivity to Pd catalysis. The *para* and *ortho* isomers **2c** and **2d**, which are designed to undergo 1,6- or 1,4-elimination upon *O*-depropargylation (ESI, Fig. S1†), were prepared to test which position provides superior protection and, consequently, a greater therapeutic window relative to the active drug (EC_50_ (**2c, d**)/EC_50_ (**1**)).

### Cytotoxicity study: drug *vs.* prodrug

The efficacy of the deactivation strategy was evaluated by treating human prostate cancer DU145 and glioma U87 cells (models of human cancers that are clinically treated with local therapies)^3^,^35^ for 5 d with increasing concentrations of **1** and **2a–d** (0.001 to 10 μM). EC_50_ values were calculated from the generated 10-point semilog dose–response curves (see ESI, Fig. S2†) and plotted in Table 1. All prodrugs showed a significant reduction in cytotoxicity relative to **1**. A direct relationship between the reduction of bioactivity and increasing steric bulk around the carbamate group was consistently observed. Derivative **2a** (containing the small Poc group) showed a relatively low difference in activity compared to **1** (30–40 fold). Notably, PBC-masked prodrugs **2c** and **2d** exhibited much greater reduction of cytotoxicity, with the *ortho* analog **2d** displaying a difference in antiproliferative activity of more than two orders of magnitude relative to unmodified drug **1** (approx. 350- and 150-fold in DU145 and U87, respectively). These results indicate that the masking group of **2d** provides superior protection to the carbamate bond against unspecific biological cleavage, even compared to the *para* analog **2c** and without the need of further increment of its size.

**Table 1:** Calculated EC_50_ values (μM) for **1** and **2a–d** treatment in DU145 and U87 cells

Cell line	DU145		U87	
Reagent	EC_50_	EC_50_ (**2**)/EC_50_ (**1**)	EC_50_	EC_50_ (**2**)/EC_50_ (**1**)
**1**	0.02	—	0.08	—
**2a**	0.83	41.5	2.49	31
**2b**	2.69	134.5	3.13	39
**2c**	5.54	277	5.27	66
**2d**	7.13	356.5	11.8	147.5

### Zebrafish cardiotoxicity study

**Fig. 1:**
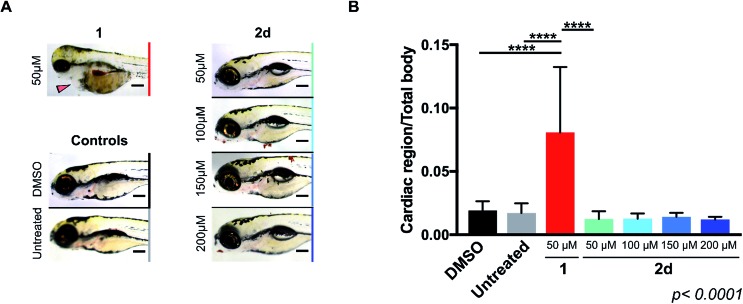
(A) Lateral view of 120 hpf zebrafish larvae after exposition to 50 μM of **1** or 50–200 μM of **2d** for 4 d. Zebrafish treated with 0.1% DMSO and E3 medium only (untreated) were used as negative controls. Cardiac edema (only observed for fish treated with **1**) is indicated with a red arrow. Additional toxicity in the eyes and the brain is also apparent. Scale bar = 20 μm. (B) Histogram displaying the ratio of cardiac area/total body area for zebrafish populations in 0.1% DMSO (black), E3 medium alone (untreated, grey), **1** at 50 μM (red) and **2d** at 50–200 μM (light to dark blue with increasing concentrations). Zebrafish populations treated with >50 μM of **1** died during treatment and were excluded from analysis. Error bars: ±SD from *n* = 3 (15 fish per biological replicate); *p* < 0.0001, **** (ANOVA).

As mentioned above, the induction of cardiotoxicity is the most severe side effect of **1**. Given the greater therapeutic window afforded by **2d**, an *in vivo* cardiotoxicity assay was designed to study its effect on the heart of developing zebrafish in comparison to **1**. Dechorionated zebrafish embryos (1 dpf) were treated for 4 days with **1** or **2d** at a range of concentrations (50–200 μM) and then imaged (brightfield and fluorescent microscopy). Embryos in E3 medium alone or with DMSO were used as negative controls. To determine the enlargement of the heart (=estimate of pericardial edema), the ratio between the cardiac area and the total body of the treated fish was measured and compared with the untreated controls. As shown in Fig. 1, treatment of **1** at 50 μM induced significant increment of the cardiac area, with 100% lethality observed in all populations treated at higher concentrations. In contrast, **2d** did not induce lethality or cardiac edema even up to 200 μM; well above the theoretical blood concentration achieved with the maximum therapeutic dosage of clinical dosing regimens.^36^ Since spectrophotometry analysis of the optical properties of **1** and **2d** showed similar red fluorescent properties (ESI, Fig. S3†), drug/prodrug distribution patterns were analyzed by fluorescent microscopy (Ex/Em = 520/560 nm). Image analysis showed that significant levels of **1** accumulated across the body of the zebrafish, including the heart area, while **2d** did not (ESI, Fig. S4†). This observation is consistent with **2d**'s lack of capacity to engage with the *in vivo* molecular targets of **1**.

### Prodrug-into-drug conversion studies

Pd-devices were prepared from amino-functionalized TentaGel® HL resins of 110 μm in average diameter (Rapp Polymere GmbH) as previously described.^20^ Content in Pd metal was determined to be 4.47% w/w by inductively coupled plasma optical emission spectrometry (ICP-OES). The conversion of inactive **2a–d** into cytotoxic **1** was first tested by performing a Pd-mediated activation assay in cancer cell culture at a single concentration, using cell viability measurements as a functional readout of the experiment. DU145 cells were treated with prodrugs **2a–d** (0.3 μM) and Pd-devices (0.6 mg mL^–1^) and cell viability determined at day 5 using the PrestoBlue® reagent. Cells treated with only Pd-devices (0.6 mg mL^–1^) or **2a–d** (0.3 μM) were used as negative controls and unmodified **1** (0.3 μM) as positive control.

**Fig. 2:**
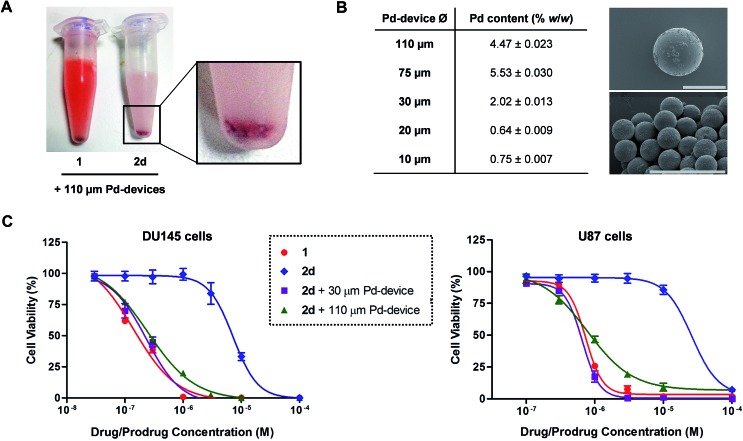
(A) Sequestration effect of prodrug **2d** by Pd-devices. 100 μM of **1** or **2d** and 1 mg mL^–1^ of 110 μm Pd-devices were mixed in PBS and stirred for 30 min. The mixtures were then frozen by immersing the tubes in liquid nitrogen and photographed. Equivalent results were observed for prodrugs **2a–c**. (B) ICP-OES determination of Pd loading in Pd-devices ranging from 110 to 10 μm in diameter. SEM images of 110 μm Pd-devices (top; ×450 magnification) and 30 μm Pd-devices (bottom; ×800 magnification). Scale bar = 100 μm. (C) Study of the activation of **2d** with 30 μm and 110 μm Pd-devices in DU145 (left) and U87 (right) cancer cell culture at a range of concentrations. Experiments: **1** (red), **2d** (blue), and **2d** + 0.6 mg mL^–1^ 30 μm Pd-devices (purple), and **2d** + 0.6 mg mL^–1^ 110 μm Pd-devices (green). Semi-log dose–response curves were generated by measuring cell viability at day 5 using PrestoBlue® reagent. Error bars: ±SD from *n* = 3.

As expected, Cbz-protected **2b** displayed no signs of toxicity either on its own or in the presence of the catalyst, which confirms its resistance to both metabolic and Pd-mediated activation (ESI, Fig. S5†). On the contrary, prodrugs **2a–2d** elicited no harm on their own but significant antiproliferative effects in the presence of the catalytic devices, thus proving the selective *in situ* generation of **1** in response to the presence of Pd. However, the assay revealed a statistically-significant gap in cytotoxicity between **1** and the prodrug/Pd-devices combinations, particularly for prodrugs **2c** and **2d**. By simply mixing the prodrugs and the Pd-devices in a test tube, it was visibly noticed that the devices had a remarkable capacity to sequester **2d** from the solution, an effect that was not seen for **1** (Fig. 2A). The polymeric component of these devices is made of TentaGel®, a copolymer consisting of a low crosslinked polystyrene (PS) matrix on which polyethylene glycol (PEG) is grafted.^44^ Based on the strong “trapping” effect observed for **2d**, we reasoned that the lipophilic polystyrene core of the devices were likely responsible for capturing the prodrug, partially deterring its effective interaction with the catalyst and, in turn, resulting in a moderate reduction of the reaction rate and subsequent liberation of **1** into the cell culture.

Aiming to optimize the functionality of the devices, we prepared, characterized and tested a range of Pd-devices using polymer resins of reduced size and greater PEG/PS ratio (=increased hydrophilicity). Pd-functionalized devices with sizes of 10, 20, 30 and 75 μm were synthesized using commercially available TentaGel® resins (Rapp Polymere GmbH) following the same protocol used for the preparation of 110 μm Pd-devices.^20^ ICP-OES analysis demonstrated the presence of the metal in each of the devices, although lower Pd content was observed in the smaller ones (Fig. 2B). The catalytic capacity of the devices was tested by 24 h treatment with nonfluorescent bis-*N*,*N*′-(propargyloxycarbonyl)rhodamine 110 (**3**) in cell culture conditions (PBS + 10% serum, pH = 7.4, 37 °C). This assay is based on the fluorogenic release of rhodamine 110 (**4**) upon Pd-mediated *O*-depropargylation of **3**. Fluorescence intensity was measured with a spectrophotometer. As shown in Fig. S5C (ESI),† the 30 μm Pd-devices displayed slightly superior catalytic activity then the rest of the devices. The catalytic properties of the devices remained stable after storage at 4 °C for 20 months (ESI, Fig. S6†). Study of the tolerability of DU145 and U87 cells to the presence of 30 μm Pd-devices showed full biocompatibility at the concentrations tested (ESI, Fig. S7†).

Prodrug candidate **2d** was then incubated with 30 μm Pd-devices in serum-free media at physiological conditions (pH = 7.4, isotonicity, 37 °C) and analyzed by UPLC. Remarkably, whereas the analysis indicated that the 30 μm devices also sequestered the prodrug very rapidly (note the immediate disappearance of the prodrug signal from the chromatogram in Fig. S8 of the ESI†), it showed efficient synthesis and release of **1**, an optimal situation for the therapeutic goal of the strategy. In principle, the superior affinity of the prodrug for the devices could potentially serve to pull the prodrug molecules out of circulation, whereby they would be uncaged and released as an active drug at the intended site of treatment. As shown in Fig. 2C, dose–response studies in cancer cell culture using 30 μm Pd-devices provided equivalent curves and EC_50_ values for the prodrug **2d**/Pd combinations and the unmodified drug **1** in both cell lines (see complete studies in ESI, Fig. S9 and S10†). Based on the safety, catalytic performance and convenient size of the 30 μm Pd-devices, they were then chosen for the next phase of the investigation.

### *In vivo*/*ex vivo* assays: implantation, safety and stability of Pd-devices

**Fig. 3:**
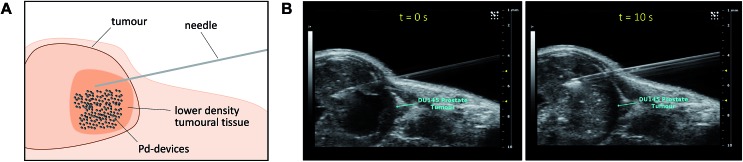
(A) Schematic of the *in vivo* intratumoural insertion of 30 μm Pd-devices (1 mg in 25 μL of PBS) into a murine prostate tumour. (B) Ultrasound image captions of the prostate tumour area of an anaesthetised mouse taken during the implantation procedure at *t* = 0 s (left) and 10 s (right).

Given the medical interest of our team in developing improved prostate cancer treatments, a state-of-the-art orthotopic murine model of human prostate cancer^45^,^46^ was used to test the *in vivo* biocompatibility and functional stability of the Pd-devices. DU145 prostate cancer cells were carefully inserted in the prostate of anesthetized male mice (*n* = 4) and tumours allowed to grow for 4 weeks (see full details in the ESI†). One of the clinical logistics of implant-based focal therapy modalities is to insert the devices in the tumour with high precision. In brachytherapy, this procedure is facilitated by the echogenic nature of the radioactive inserts (which are made of metal), as it enables to control the correct placement of the radioactive pellets using ultrasound imaging.^47^ Since the Pd-devices contain a significant amount of metallic material, we envisioned that ultrasound-guided insertion would be an optimal method to place such small devices with millimeter range precision in the prostate tumour (Fig. 3A). A standard 1 mL syringe fitted with a 9-gauge needle was used to deliver the devices. The size of the 30 μm Pd-devices was found optimal to perform this procedure with ease, whereas partial blockage of the needle was observed with the larger devices. Hence, 1 mg of 30 μm Pd-devices suspended in 25 μL of sterile PBS was injected into the prostate tumour of anaesthetized mice under real time monitoring with ultrasound imaging. Fig. 3B and Movie S1† shows the highly echogenic Pd-devices being precisely inserted into a hypoechogenic area of the tumour. The mice were then transferred for recovery. The injected region was imaged 30 min after the procedure (Movie S2†) and monitored for 3 days (ESI, Fig. S11†) to ensure that Pd-devices did not leak out the tumour. Tumour growth was not affected by the presence of the devices. Apart from the presence of the tumour, animals remained healthy and behaved normally during the time of the study. After 21 days, the animals were sacrificed. Necropsy of animals and gross observation of major organs showed no macroscopic signs of toxicity. Tumours were removed and sliced in half to study the distribution of the Pd-devices (ESI, Fig. S11†). Examination of the tissue surrounding the injection site further confirmed that the devices had not been displaced from the cancerous tissue.

**Fig. 4:**
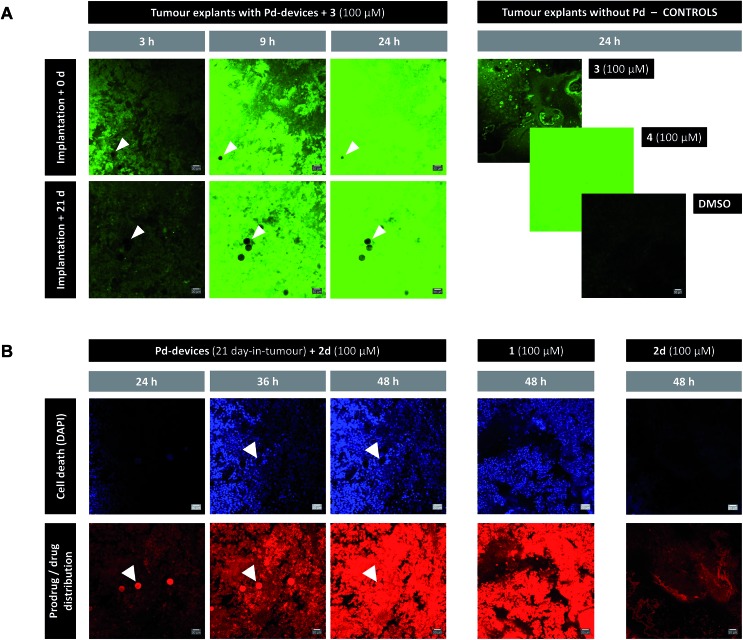
(A) Live-cell imaging study of the catalytic activity of tumour-implanted Pd-devices: 0 d *vs.* 21 d after implantation. Pd-bearing explants (*n* = 2) were incubated with **3** (100 μM) and imaged by live-cell confocal microscopy (488 laser excitation) at 3, 9 and 24 h. The presence of the Pd-devices is indicated with white arrows. Note that Pd-devices are identified due to their capacity to quench green fluorescence.^27^ Images of the controls (**3** alone (100 μM); **4** alone (100 μM); and DMSO) are shown at 24 h. Pictures were generated using ImageJ software. Scale bar = 30 μm. (B) Live-cell imaging study of the Pd-mediated conversion of inactive **2d** into cytotoxic **1** in a human prostate tumour explant model. Images of a representative tumour explant (*n* = 2) bearing 21 day-in-tumor Pd-devices after treatment with **2d** (100 μM) in the presence of DAPI for 24, 36 and 48 h (left panel). Tissue samples were imaged under laser excitation at 405 nm (for DAPI staining, in blue) and 543 nm (for **2d**/**1** distribution, in red). The presence of the Pd-devices, indicated with white arrows, is identified by a bright fluorescence signal in the red channel (as previously reported,^48^ red fluorescence is not quenched by Pd). Images of the Pd-free controls **1** (100 μM, mid panel) and **2d** (100 μM, right panel) are shown at 48 h. Pictures were generated using ImageJ software. Scale bar = 30 μm.

The catalytic capacity of Pd-devices hosted in the prostate tumour of live mice for 3 weeks (21 day-in-tumour Pd-devices) was then tested *ex vivo*. Explants bearing Pd-devices were sliced longitudinally using a rat brain slicer instrument into 1 mm sections, soaked in media containing fluorogenic probe **3** and visualized by live-cell imaging confocal microscopy for 24 h (using the green fluorescent channel with 488 nm laser excitation). The catalytic activity of 21 day-in-tumor Pd-devices was compared with devices directly implanted in the explants (0 day-in-tumour Pd-devices). Incubation of Pd-free explants with **3**, **4** or DMSO were used as controls. As shown in Fig. 4A, a rapid increase in fluorescent intensity (=release of fluorescent dye **4**) was achieved by treating Pd-bearing explants with **3**. Importantly, equivalent fluorescence generation was observed with both 0- and 21 day-in-tumour Pd-devices, evidence that maintaining the devices in living tissue for extended periods of time does not have a negative impact in the underlying turnover rate of the devices. In contrast, in the absence of the activation devices, tumour explants treated with **3** showed minimal background signal (Fig. 4A, right). The good biocompatibility and catalytic stability of the catalyst in living tissue, which are herein shown for the first time for a bioorthogonal catalyst that has been hosted for weeks in a tumour *in vivo*, are optimal features that support the use of heterogeneous Pd devices for site-specific prodrug activation in precision medicine.

### *Ex vivo* prodrug-into-drug conversion study

Next, the capacity of the devices to generate therapeutic (=cytotoxic) levels of **1** from an inactive dose of **2d** was tested in tumour explants bearing 21 day-in-tumour Pd-devices. Tissue samples were treated for 48 h in media containing 100 μM of **2d** in the presence of DAPI (a nuclei stain not permeable in viable cells). Treatment of Pd-free explants (in the presence of DAPI) with either **2d** (100 μM) or **1** (100 μM) were used as a negative and positive control, respectively. Cancer cell viability and prodrug/drug distribution was monitored by live-cell imaging confocal microscopy using two laser excitations: 405 nm to image nuclei staining by DAPI (=dying/dead cells) and 543 nm to visualize **2d** and/or **1** (the spectroscopic properties of both compounds are indistinguishable; see Fig. S3†). In the absence of Pd-devices (Fig. 4B, right panel), 48 h treatment with **2d** did not elicit any sign of cell death in the cancer tissue. Analysis of the red fluorescent channel shows that **2d** was not retained inside the cancer cells, which is consistent with its expected inability to bind with the intracellular targets of doxorubicin. A drastically different distribution profile was observed upon treatment with **1**, with bright red fluorescence being accumulated in the cells (Fig. 4B, middle panel; and ESI, Fig. S12†). Remarkably, the distribution pattern of the red fluorescence signal from explants bearing Pd-devices and treated with **2d** became identical to that of the explants directly treated with **1** after 48 h, indicating that **2d** has been chemically converted into **1**. It is also noteworthy that compound **2d** rapidly entered the devices during the first hours of treatment, making the Pd-devices patently visible under the 543 nm laser excitation (Fig. 4B, see panels at 24 and 36 h). This observation agrees with the high capacity of the devices to “pull” the prodrug from the environment (Fig. 2A). Importantly, the **2d**/Pd-devices treatment combination induced a strong cytotoxic effect (DAPI staining), equivalent to that of **1** treatment, demonstrating the *in situ* bioorthogonal generation of bioactive levels of drug. *Ex vivo* studies with the 0 day-in-tumour Pd-devices provided equivalent results (ESI, Fig. S12†).

Last, samples of an explant containing 21 day-in-tumour Pd-devices and previously treated with fluorogenic compound **3** were washed and subsequently incubated with either **1**, prodrug **2d** or DMSO for 48 h. Tissue samples were then fixed and analysed by immunohistochemical staining with caspase 3 (apoptosis biomarker) or γ-H2AX (biomarker for DNA double-strand breaks) antibodies (ESI, Fig. S13†). As expected, damaged cells (indicated by brown staining) were only observed in the Pd-containing samples treated with **1** and **2d**. This test agrees with the inert nature of the devices and supports their capacity to activate multiple doses of prodrug.

## Conclusions

In conclusion, fundamental insights into the validity, reliability and clinical feasibility of a novel approach to accurately target chemotherapy spatially within a tumour have been reported. This therapeutic approach, which is conceived for the treatment of localised cancers, consists of two components: (i) an inactive derivative of a cytotoxic drug designed to be specifically activated by palladium chemistry; and (ii) an inert implantable polymer-based device functionalised with Pd nanoparticles to catalyse drug conversion and release in a spatially controlled manner. As part of the investigation, a novel caged doxorubicin was developed by blocking the NH_2_ group of the drug's sugar moiety with a Pd-labile PBC group. The derivative having the propargyloxy moiety in *ortho* was found to offer superior reduction of bioactivity than the one in *para* and high sensitivity to Pd catalysis. Studies in zebrafish proved that the cardiotoxic effect induced by doxorubicin was abolished in the caged precursor; a clinically-relevant result that to the best of our knowledge is shown in this animal model for the first time for a doxorubicin prodrug. Pd-devices of optimised size and activity were also developed and tested in a state-of-the-art orthotopic murine model of human prostate cancer. The devices showed high echogenicity, enabling precise injection into the tumour using ultrasound scanning, an image-guided microsurgical technique widely used in the clinic. The study provided evidence of the non-perturbing effect of the devices to the mice and of the resilience and biocompatibility of the catalyst to the tumour microenvironment. Devices hosted in a tumour of mice for 21 d elicited catalytic release of a fluorogenic probe and a caged doxorubicin at an equivalent rate than freshly injected devices. *Ex vivo* time-lapse imaging enabled to monitor the distinct distribution profiles of the drug and the inactive precursor, and the capacity of the devices to “sequester” circulating prodrug molecules and generate cytotoxic levels of drug in the tumour explant. This investigation provides compelling evidence that heterogeneous palladium catalysts can be applied as bioorthogonal tools to enable local treatment of disease and reports a standardized preclinical methodology to perform and study local bioorthogonal release of bioactive substances in preclinical disease models.

## Conflicts of interest

The authors declare that compounds **2c, d** are protected under patent application PCT/GB2017/051379.

## Supplementary Material

Supplementary information

Supplementary movie

Supplementary movie
