# Supplementary material for: Bright insights into palladium-triggered local chemotherapy
Source: Chem Sci. 2018 Jul 17;9(37):7354–61. doi: 10.1039/c8sc02291g (PMC6237126; doi:10.1039/c8sc02291g)
Supplement: Supplementary file 1 [file SC-009-C8SC02291G-s001.pdf]

Electronic Supplementary Material (ESI) for Chemical Science.
This journal is © The Royal Society of Chemistry 2018

Electronic Supplementary Information

Bright insights into Pd-triggered local chemotherapy

Thomas L. Bray,[a] Mark Salji,[bc] Alessandro Brombin,[ad] Ana M. Pérez-López,[a] Belén Rubio-Ruiz,[a] Laura Galbraith,[bc] E. Elizabeth Patton,[ad] Hing Y. Leung,[bc,*] Asier Unciti-Broceta[a,*]

[a]*Cancer Research UK Edinburgh Centre and* [b]*MRC Human Genetics Unit, Institute of Genetics & Molecular Medicine, University of Edinburgh, Crewe Road South, Edinburgh EH4 2XR, UK;* [c]*Institute of Cancer Sciences, University of Glasgow, Bearsden, Glasgow G61 1QH, UK;* [d]*CRUK Beatson Institute, Bearsden, Glasgow G61 1BD, UK*

LIST OF CONTENTS

1. General experimental protocols 2

2. Synthesis and characterisation of compounds **2a-d** 3-9

3. Synthesis and characterisation of **Pd-devices** 10

   3.1. Synthesis protocol 10

   3.2. Scanning Electron Microscopy imaging 10

   3.3. Inductively Coupled Plasma-Optical Emission spectrometry 10

4. Non-biological conversion studies 11

   4.1. Non-biological Pd mediated activation of fluorogenic probe **3** 11

   4.2. Non-biological Pd mediated prodrug-into-drug conversion study 11

5. Biological Methods 12

   5.1. Cell culture 12

   5.2. Cytotoxicity study: drug vs prodrug 12

   5.3. Prodrug-into-drug conversion studies 12

6. Zebrafish toxicology study 13

7. In vivo / ex vivo studies 14

   7.1. Intratumoural ultrasound-guided implantation of Pd-devices 14

   7.2. Ex vivo catalytic studies 14

8. References 15

9. Supplementary Figures S1-S13 16-28

1. General experimental protocols

All chemicals were purchased from Sigma Aldrich, VWR International Ltd or Fisher Scientific UK Ltd unless otherwise stated. Doxorubicin HCl was purchased from Cambridge Biosciences Ltd.

Compounds were purified by manual flash column chromatography with commercially available silica gel (220 – 440 mesh, Sigma-Aldrich). Columns and solvents used to elute and purify the products varied depending on the amount of starting material used and polarity of compounds. R_f values were determined on Merck aluminium backed TLC Silica gel 60 F254 sheets. Compounds were visualised using a UV6L-58 Mineralight UV emitter at 254 nm.

Ultra-performance liquid chromatography (UPLC) was performed using a Waters Acquity UPLC PDA with a 50 x 2.4 mm Waters BEH C18 1.7 µm column, with compounds detected using an evaporative light scattering detector (ELSD) and UV detector. All solvents used were HPLC grade. Method: eluent A, H_2O and formic acid (0.1 %); eluent B, acetonitrile; A/B = 95 : 5 to 20 : 80 in 8 min, 95 : 5 in 8.5 min. Compounds used in the biological screenings were determined to be >95% pure by analytical HPLC with evaporative light scattering detection (Agilent).

^{1}H and ^{13}C NMR were recorded at ambient temperature on Bruker Avance III 500 Mhz or 600 Mhz spectrometers from the Edinburgh University School of Chemistry. All chemical shifts are reported relative to the solvent peak in parts per million (δ). High Resolution Mass Spectra (HRMS) were obtained using a Bruker 3.0 T Apex II Spectrometer under electron spray ionisation conditions.

2. Synthesis and characterization of compounds 2a-d

2.1. Synthesis of Pd-labile masking groups

Synthesis of propargyloxybenzyl alcohol derivatives. Potassium carbonate (4.7 g) was added to a solution of *o*-hydroxybenzyl alcohol or *p*-hydroxybenzyl alcohol (2.5 g) dissolved in acetonitrile (50 mL). After stirring at room temperature for 1 h, the mixture was treated dropwise with propargyl bromide (2.93 mL as 80 % (v/v) in toluene) and heated to reflux for 48 h. The solution was then filtered, concentrated *in vacuo*, and the crude was purified via flash chromatography (30 % ethyl acetate in hexane).

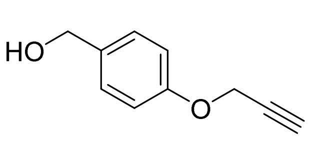

p-Propargyloxybenzyl alcohol. The synthetic method described using *p*-hydroxybenzyl alcohol gave a viscous bright yellow liquid (3.7 g, 99 %). R_f = 0.36 (30 % ethyl acetate in hexane). ^{1}H NMR (500 MHz, $CDCl_3$) δ 7.19 (d, *J* = 8.8 Hz, 2H), 6.93 – 6.85 (m, 2H), 4.60 (d, *J* = 2.6 Hz, 2H), 4.44 (s, 2H), 3.73 (s, 1H), 2.55 (t, *J* = 2.4 Hz, 1H). ^{13}C NMR (126 MHz, $CDCl_3$) δ 156.6, 133.9, 128.3 (CH), 114.6 (CH), 78.5, 75.7 (CH), 63.9 (CH_2), 55.6 (CH_2). HRMS (m/z): $[M+Na]^+$ calcd for $C_{10}H_{10}O_2$ $[M+Na]^+$: 185.0573, found: 185.0576.

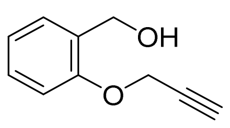

o-Propargyloxybenzyl alcohol. The synthetic method described using *o*-hydroxybenzyl alcohol gave a viscous dark yellow liquid (1.0 g, 30 %). R_f = 0.37 (30 % ethyl acetate in hexane). ^{1}H NMR (500 MHz, $CDCl_3$) δ 7.34 (dd, *J* = 7.5, 1.7 Hz, 1H), 7.28 – 7.22 (m, 1H), 6.99 (td, *J* = 7.4, 0.9 Hz, 1H), 6.94 (d, *J* = 8.3 Hz, 1H), 4.68 – 4.61 (m, 4H), 3.42 (s, 1H), 2.57 (t, *J* = 2.4 Hz, 1H). ^{13}C NMR (126 MHz, $CDCl_3$) δ 154.8, 129.7 (CH), 128.4, 128.2 (CH), 121.3 (CH), 111.6 (CH), 78.5, 75.7 (CH), 60.3 (CH_2), 55.7 (CH_2). HRMS (m/z): $[M+Na]^+$ calcd for $C_{10}H_{10}O_2$ $[M+Na]^+$: 185.0573, found: 185.0573.

Synthesis of p-nitrophenyl carbonate derivatives. A solution of 4-nitrophenylchloroformate (0.48 g) in dry DCM (8 mL) was added dropwise to a solution of the alcohol (2.2 mmol) and pyridine (0.19 mL) in DCM (8 mL) at 0 °C under nitrogen in the dark. The mixture was stirred from 0 °C to room temperature overnight and monitored by to indicate full consumption of the alcohol (at approx. 20 h). After concentrating *in vacuo,* the crude residue was re-dissolved in ethyl acetate (70 mL), washed with H_2O (2 x 50 mL) and brine (2 x 50 mL), dried over $MgSO_4$ and re-concentrated *in vacuo,* and the crude was purified via flash chromatography (50 % DCM in hexane).

p-Nitrophenyl propargyl carbonate. The synthetic method described using propargyl alcohol gave white crystals (0.31 g, 64 %). R_f = 0.29 (50 % DCM in hexane). ^{1}H NMR (500 MHz, $CDCl_3$) δ 8.32 – 8.25 (m, 2H), 7.44 – 7.38 (m, 2H), 4.88 (d, *J* = 2.5, 2H), 2.62 (t, *J* = 2.5, 1H). ^{13}C NMR (126 MHz, $CDCl_3$) δ 155.5, 152.1, 145.7, 125.5 (CH), 121.9 (CH), 77.4, 76.9 (CH), 56.6 (CH_2). HRMS (m/z): $[M+Na]^+$ calcd for $C_{10}H_7O_5N_1$ $[M+Na]^+$: 244.0216, found: 244.0206.

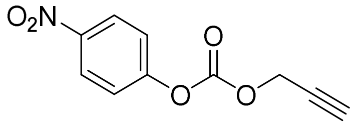

p-Nitrophenyl benzyl carbonate. The synthetic method described using benzyl alcohol gave white crystals (0.41 g, 68 %). R_f = 0.34 (50 % DCM in hexane). ^{1}H NMR (500 MHz, $CDCl_3$) δ 8.30 – 8.26 (m, 2H), 7.48 – 7.36 (m, 7H), 5.30 (s, 2H). ^{13}C NMR (126 MHz, $CDCl_3$) δ 155.6, 152.6, 145.5, 134.3, 129.2 (CH), 129.0 (CH), 128.9 (CH), 125.5 (CH), 121.9 (CH), 71.2 (CH_2). HRMS (m/z): $[M+Na]^+$ calcd for $C_{14}H_{11}O_5N_1$ $[M+Na]^+$: 296.0529, found: 296.0507.

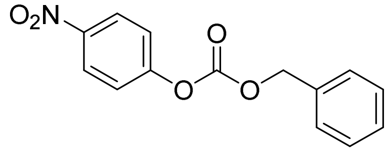

p-Nitrophenyl p-propargyloxybenzyl carbonate. The synthetic method described using *p*-propargyloxybenzyl alcohol provided a white oil that solidified to a white solid when below 20 °C (0.54 g, 75 %). R_f = 0.20 (50 % DCM in hexane). ^{1}H NMR (500 MHz, $CDCl_3$) δ 8.28 – 8.24 (m, 2H), 7.42 – 7.38 (m, 2H), 7.38 – 7.35 (m, 2H), 7.03 – 6.98 (m, 2H), 5.24 (s, 2H), 4.71 (d, *J* = 2.4 Hz, 2H), 2.53 (t, *J* = 2.4 Hz, 1H). ^{13}C NMR (126 MHz, $CDCl_3$) δ 158.3, 155.7, 152.6, 145.5, 130.7, 127.4 (CH_2), 125.4 (CH_2), 121.9 (CH_2), 115.3 (CH_2), 78.4, 75.9 (CH), 70.9 (CH_2), 56.0 (CH_2). HRMS (m/z): $[M+Na]^+$ calcd for $C_{17}H_{13}O_6N_1$ $[M+Na]^+$: 350.0635, found: 350.0646.

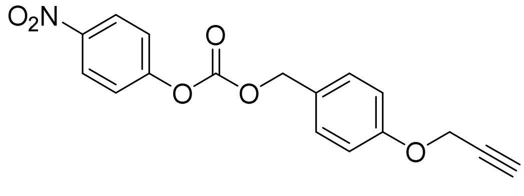

p-Nitrophenyl o-propargyloxybenzyl carbonate. The synthetic method described using *o*-propargyloxybenzyl alcohol provided a white oil that solidified to a white solid when kept below 20 °C (0.52 g, 72 %). R_f = 0.19 (50 % DCM in hexane). ^{1}H NMR (500 MHz, $CDCl_3$) δ 8.28 – 8.23 (m, 2H), 7.44 – 7.36 (m, 4H), 7.08 – 7.01 (m, 1H), 5.39 (s, 1H), 4.78 (d, *J* = 2.4 Hz, 1H), 2.53 (t, *J* = 2.4 Hz, 1H). ^{13}C NMR (126 MHz, $CDCl_3$) δ 155.9, 155.8, 152.5, 145.4, 130.6 (CH), 130.5, 125.4 (2CH), 123.4 (CH), 121.9 (CH), 121.7 (CH), 112.4 (CH), 78.4, 76.0 (CH), 66.6 (CH_2), 56.3 (CH_2). HRMS (m/z): $[M+Na]^+$ calcd for $C_{17}H_{13}O_6N_1$ $[M+Na]^+$: 350.0635, found: 350.0590.

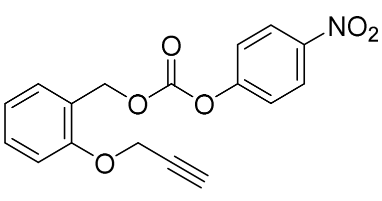

2.2. Synthesis of compounds 2a-d

General Procedure. A solution of the corresponding alkyl p-nitrophenyl carbonate (55.2 μmol) in anhydrous DMF (2 mL) was flushed with nitrogen after stirring for 10 min, then syringed into a flask containing a solution of doxorubicin HCl (20 mg) and triethylamine (7.5 μL) in anhydrous DMF under nitrogen at room temperature. The reaction was monitored by TLC (10 % methanol in DCM) to observe complete consumption of doxorubicin. After 20 h, the reaction was diluted with H_2O (50 mL) and extracted with ethyl acetate (4 x 50 mL). The combined organic extracts were concentrated down to a volume of ~100 mL, then washed successively with saturated $NaHCO_3$ (2 x 50 mL), H_2O (2 x 50 mL) and brine (2 x 50 mL), dried over $MgSO_4$, concentrated *in vacuo* with the water bath kept below 40 °C and the crude was purified via flash chromatography (0 → 2 % methanol in DCM).

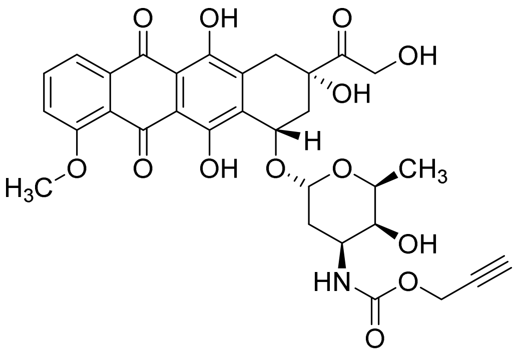

*N-Propargyloxycarbonyl doxorubicin (**2a**).* The synthetic method described using *p*-nitrophenyl propargyl carbonate gave a dark red powder (12.4 mg, 73 %). R_f = 0.25 (2 % methanol in DCM). ^{1}H NMR (500 MHz, $CDCl_3$) δ 13.96 (s, 1H), 13.21 (s, 1H), 8.03 (dd, *J* = 7.7, 1.0 Hz, 1H), 7.81 – 7.74 (m, 1H), 7.39 (d, *J* = 8.0 Hz, 1H), 5.50 (d, *J* = 3.8 Hz, 1H), 5.28 (s, 1H), 5.21 (d, *J* = 8.5 Hz, 1H), 4.76 (d, *J* = 1.8 Hz, 2H), 4.61 (d, *J* = 2.2 Hz, 2H), 4.51 (s, 1H), 4.14 (q, *J* = 6.3 Hz, 1H), 4.08 (s, 3H), 3.91 – 3.83 (m, 1H), 3.67 (s, 1H), 3.26 (dd, *J* = 18.8, 1.7 Hz, 1H), 2.99 (d, *J* = 18.8 Hz, 2H), 2.42 (d, *J* = 2.1 Hz, 1H), 2.33 (d, *J* = 14.7 Hz, 1H), 2.17 (dd, *J* = 14.7, 4.1 Hz, 1H), 1.88 (dd, *J* = 13.5, 5.1 Hz, 1H), 1.78 (td, *J* = 13.3, 4.2 Hz, 1H), 1.29 (d, *J* = 6.6 Hz, 3H), 1.25 (s, 1H). ^{13}C NMR (126 MHz, $CDCl_3$) δ 214.0, 187.3, 186.8, 161.2, 156.3, 155.8, 154.8, 135.9 (CH), 135.7, 133.7, 121.0, 120.0, 118.6 (CH), 111.8, 111.6, 100.8 (CH), 78.2 (CH), 77.4 (CH), 76.8, 74.8, 69.8 (CH), 69.7 (CH), 67.4 (CH), 65.7 (CH_2), 56.8 (CH_3), 52.7 (CH_2), 47.3 (CH), 35.8 (CH_2), 34.2 (CH_2), 30.5 (CH_2), 17.0 (CH_3). HRMS (m/z): $[M+Na]^+$ calcd for $C_{31}H_{31}O_{13}N_1Na_1$ 648.1687; found 648.1677.

*N-Benzyloxycarbonyl doxorubicin (**2b**).* The synthetic method described using *p*-nitrophenyl-benzyl carbonate gave a dark red powder (17.3 mg, 94 %). R_f = 0.25 (2 % methanol in DCM). ^{1}H NMR (500 MHz, $CDCl_3$) δ 13.96 (s, 1H), 13.23 (s, 1H), 8.03 (dd, *J* = 7.7, 1.0 Hz, 1H), 7.78 (dd, *J* = 8.3, 7.9 Hz, 1H), 7.39 (dd, *J* = 8.6, 0.7 Hz, 1H), 7.34 – 7.26 (m, 5H), 5.50 (d, *J* = 3.9 Hz, 1H), 5.28 (s, 1H), 5.14 (d, *J* = 8.4 Hz, 1H), 5.03 (s, 2H), 4.82 – 4.70 (m, 2H), 4.54 (s, 1H), 4.14 (dd, *J* = 13.6, 7.2 Hz, 1H), 4.08 (s, 3H), 3.92 – 3.82 (m, 1H), 3.67 (s, 1H), 3.27 (dd, *J* = 18.8, 1.9 Hz, 1H), 3.00 (d, *J* = 18.8 Hz, 2H), 2.33 (d, *J* = 14.7 Hz, 1H), 2.19 – 2.14 (m, 1H), 1.88 (dd, *J* = 13.5, 4.8 Hz, 1H), 1.77 (td, *J* = 13.2, 4.1 Hz, 1H), 1.29 (d, *J* = 6.6 Hz, 3H), 1.25 (s, 1H). ^{13}C NMR (126 MHz, $CDCl_3$) δ 214.0, 187.3, 186.9, 161.2, 156.3, 155.8, 155.7, 136.5, 135.9 (CH), 135.7, 133.7, 128.7, 128.3 (CH), 121.0, 120.0 (CH), 118.6 (CH), 111.8, 111.6, 100.9 (CH), 77.4, 76.8 (CH), 69.8 (CH), 69.7 (CH), 67.4 (CH), 66.9 (CH), 65.7 (CH_2), 56.8 (CH_3), 47.1 (CH), 35.8 (CH_2), 34.2 (CH_2), 31.1 (CH_2), 17.0 (CH_3). HRMS (m/z): $[M+Na]^+$ calcd for $C_{35}H_{35}O_{13}N_1Na_1$ 700.2001; found 700.1988.

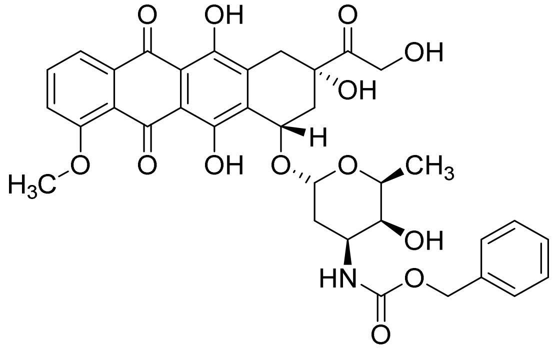

*N-(p-Propargyloxybenzyloxycarbonyl) doxorubicin (**2c**).* The synthetic method described using *p*-nitrophenyl *p*-propargyloxybenzyl carbonate gave a bright red clumpy powder (17.3 mg, 64 %). R_f = 0.25 (2 % methanol in DCM). ^{1}H NMR (500 MHz, $CDCl_3$) δ 13.96 (s, 1H), 13.23 (s, 1H), 8.03 (dd, *J* = 7.7, 1.0 Hz, 1H), 7.81 – 7.76 (m, *J* = 8.3, 7.8 Hz, 1H), 7.39 (dd, *J* = 8.5, 0.7 Hz, 1H), 7.24 (s, 1H), 6.92 (d, *J* = 8.0 Hz, 2H), 5.49 (d, *J* = 3.9 Hz, 1H), 5.28 (s, 1H), 5.10 (d, *J* = 8.3 Hz, 1H), 4.97 (s, 2H), 4.76 (s, 2H), 4.66 (s, 2H), 4.53 (s, 1H), 4.17 – 4.10 (m, 1H), 4.08 (s, 3H), 3.91 – 3.80 (m, 1H), 3.66 (s, 1H), 3.27 (dd, *J* = 18.8, 1.8 Hz, 1H), 3.05 – 2.97 (m, 2H), 2.49 (t, *J* = 2.4 Hz, 1H), 2.33 (dt, *J* = 14.5, 1.8 Hz, 1H), 2.17 (dd, *J* = 14.7, 4.0 Hz, 1H), 1.93 (d, *J* = 4.2 Hz, 1H), 1.87 (dd, *J* = 13.5, 4.8 Hz, 1H), 1.76 (td, *J* = 13.3, 4.2 Hz, 1H), 1.28 (d, *J* = 6.6 Hz, 3H), 1.25 (s, 1H). ^{13}C NMR (126 MHz, $CDCl_3$) δ 214.0, 187.3, 186.9, 161.2, 157.6, 156.3, 155.8, 157.7, 135.9 (CH), 135.7, 133.7, 134.0, 130.1 (CH), 129.6, 121.1, 120.0 (CH), 118.6 (CH), 115.1, 111.8, 111.8, 100.9 (CH), 78.6 (CH), 77.4 (CH), 76.8, 75.7, 69.8 (CH), 69.7 (CH), 67.4 (CH), 66.6 (CH), 65.7 (CH_2), 56.8 (CH_3), 55.9 (CH_2), 47.1 (CH), 35.8

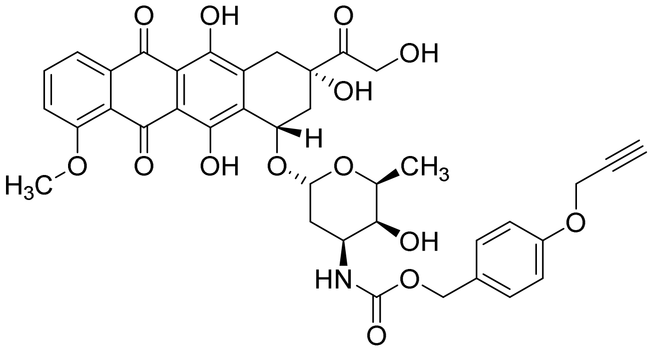

(CH_2), 34.2 (CH_2), 30.0 (CH_2), 17.0 (CH_3) HRMS (m/z): $[M+Na]^+$ calcd for $C_{38}H_{37}O_{14}N_1Na_1$ 754.2106; found 754.2130.

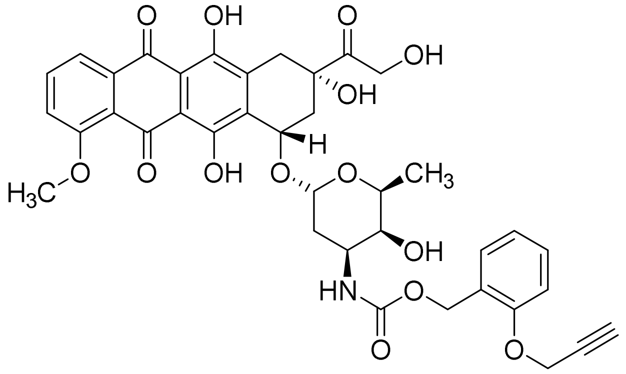

N-(o-Propargyloxybenzyloxycarbonyl) doxorubicin (***2d***). The synthetic method described using *p*-nitrophenyl *o*-propargyloxybenzyl carbonate gave a dark red clumpy powder (12.1 mg, 45 %). R_f = 0.25 (2 % methanol in DCM). ^1H NMR (500 MHz, $CDCl_3$) δ 13.98 (s, 1H), 13.25 (s, 1H), 8.04 (dd, *J* = 7.7, 1.0 Hz, 1H), 7.82 – 7.75 (m, *J* = 8.4, 7.8 Hz, 1H), 7.39 (dd, *J* = 8.5, 0.7 Hz, 1H), 7.33 – 7.27 (m, 1H), 7.01 – 6.93 (m, 2H), 5.51 (d, *J* = 3.9 Hz, 1H), 5.33 – 5.27 (m, 1H), 5.15 – 5.08 (m, 3H), 4.76 (s, 2H), 4.70 (s, 2H), 4.55 (s, 1H), 4.14 (dd, *J* = 12.3, 6.2 Hz, 1H), 4.08 (s, 3H), 3.92 – 3.82 (m, 1H), 3.68 (s, 1H), 3.28 (dd, *J* = 18.8, 1.5 Hz, 1H), 3.03 (d, *J* = 18.8 Hz, 2H), 2.48 (s, 1H), 2.34 (d, *J* = 14.7 Hz, 1H), 2.17 (dd, *J* = 14.7, 4.0 Hz, 1H), 1.88 (dd, *J* = 13.5, 5.0 Hz, 1H), 1.77 (td, *J* = 13.3, 4.2 Hz, 2H), 1.29 (d, *J* = 6.6 Hz, 3H), 1.25 (s, 1H). ^{13}C NMR (126 MHz, $CDCl_3$) δ 214.0, 187.3, 186.9, 161.2, 156.3, 155.9, 155.9, 155.6, 135.9 (CH), 135.7, 133.7, 130.0 (CH), 129.5 (CH), 125.6 (CH), 121.6, 121.1 (CH), 120.0 (CH), 118.6 (CH), 112.3, 111.8, 111.6, 111.5 (CH), 100.9 (CH), 78.7, 77.4 (CH), 76.8, 75.7, 69.8 (CH), 69.7 (CH), 67.4 (CH), 65.7 (CH), 62.2 (CH_2), 56.9 (CH_3), 56.3 (CH_2), 47.1 (CH), 35.8 (CH_2), 34.2 (CH_2), 30.1 (CH_2), 17.0 (CH_3). HRMS (m/z): $[M+Na]^+$ calcd for $C_{38}H_{37}O_{14}N_1Na_1$ 754.2106; found 754.2130.

^{1}H NMR spectra of 2c

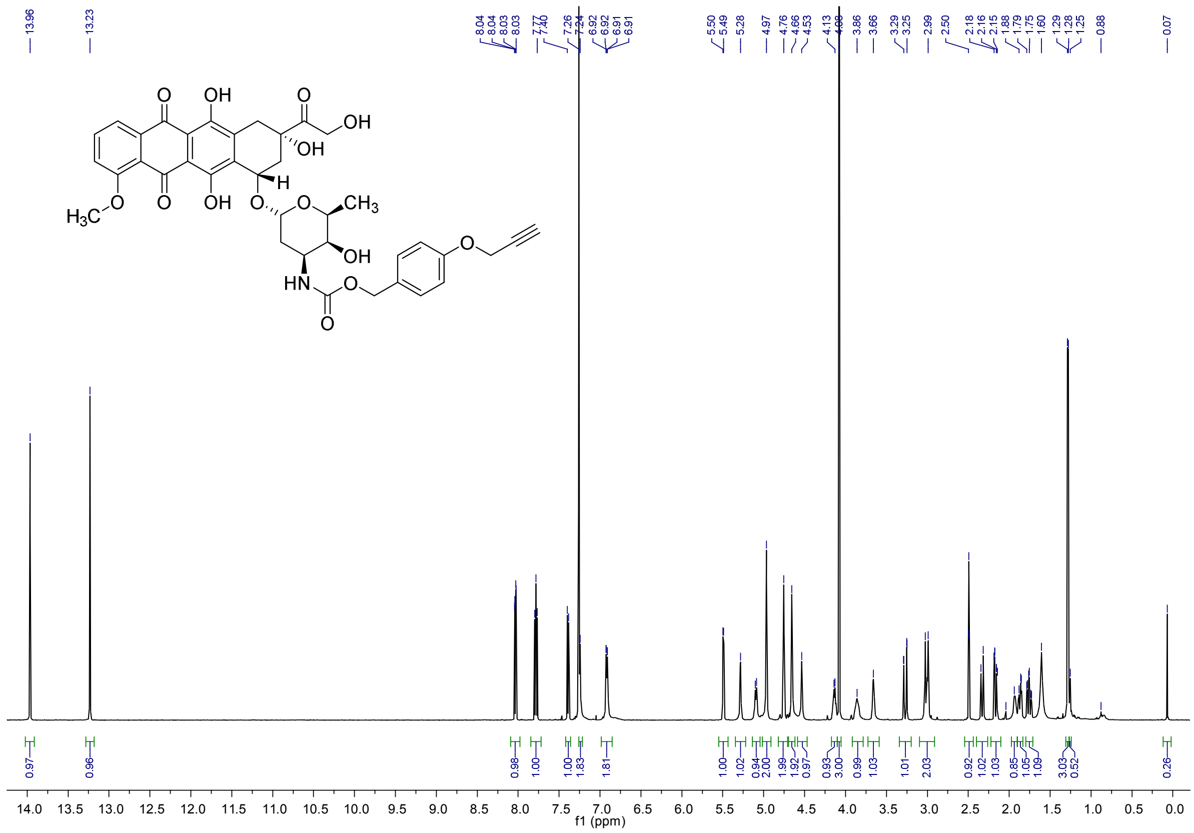

^{13}C NMR spectra of 2c

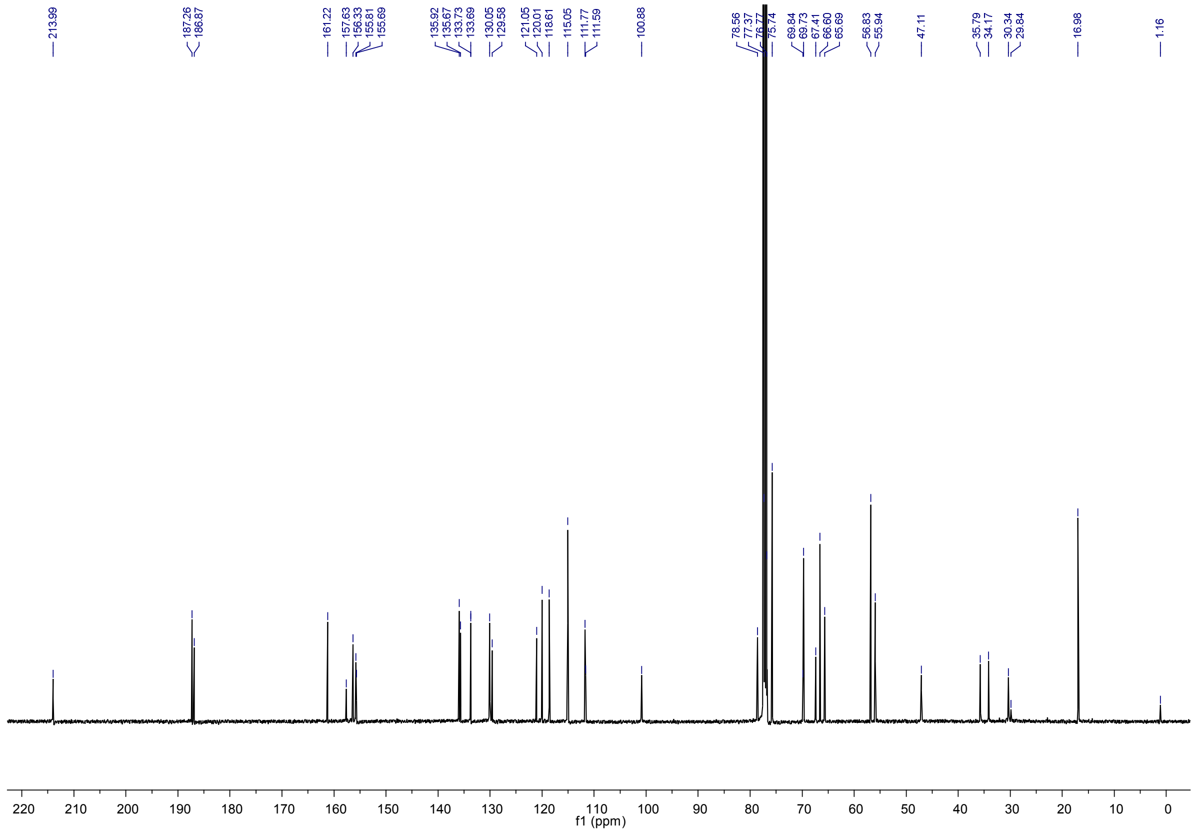

^{1}H NMR spectra of 2d

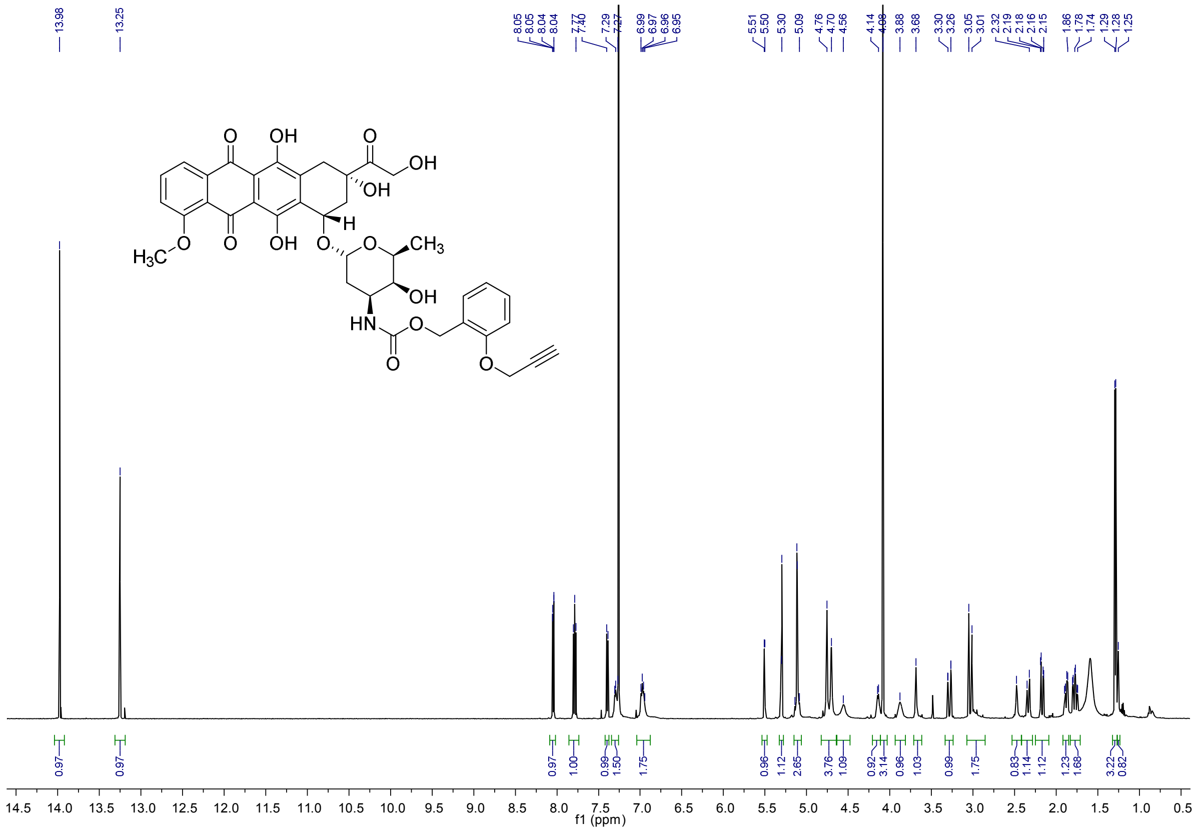

^{13}C NMR spectra of 2d

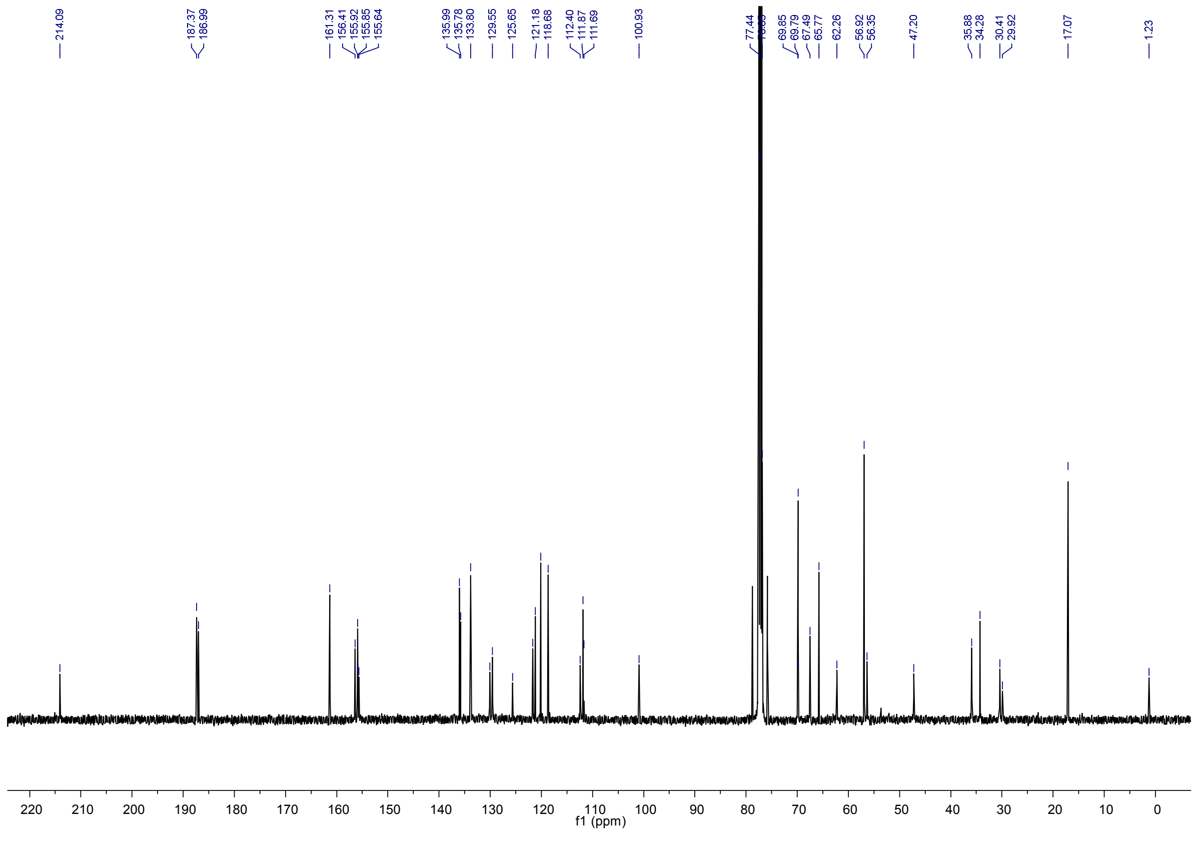

3. Synthesis and characterization of Pd-devices

3.1. Synthesis protocol

NovaSyn TG amino resins HL of variable diameter (10 µm, 20 µm, 30 µm, 75 µm and 110 µm) (1,000 mg, 0.2-0.6 NH_2 mmol/g) and palladium acetate (263 mg, 1.17 mmol) were added into a 25 mL Biotage microwave vial (Biotage). Next, toluene (10 mL) was added to the vial and heated to 80 °C under stirring for 10 min. The mixture was then stirred at room temperature for an additional 2 h. The resins were filtered and washed with dichloromethane (DCM) (5 x 20 mL) and methanol (MeOH) (5 x 20 mL). Resins were dispersed in 10 % hydrazine monohydrate in MeOH (10 mL) and stirred at room temperature for 25 min. The resins were again filtered and washed with MeOH (5 x 20 mL) and DCM (5x 20 mL). Resins were added to a solution of Fmoc-Glu(OH)-OH (216 mg, 0.59 mmol), Oxyma (166 mg, 1.17 mmol), N,N'-diisopropylcarbodiimide (DIC) (148 mg, 1.17 mmol) with DCM/dimethylformamide (DMF) (2 : 1, 9 mL) and stirred for 2 hr at rt. The resins were filtered and washed with DCM (5 x 20 mL), MeOH (5 x 20 mL) and H_2O (5 x 20 mL) and dried in an oven at 40 °C for 3 d. Completed coupling was verified by ninhydrin test after the last MeOH wash.

3.2. Scanning Electron Microscopy imaging

SEM images were performed at the School of Biological Sciences of the University of Edinburgh. Samples were fixed in a solution of 3 % glutaraldehyde in 0.1 M sodium cacodylate buffer (pH 7.3) for 2 h, then washed in 3 x 10 min changes of 0.1 M sodium cacodylate buffer. Samples were then fixed in 1 % osmium tetroxide in 0.1 M sodium cacodylate buffer for 45 min. A further three 10 min washes were performed in 0.1 M sodium cacodylate buffer. Dehydration in graded concentrations of acetone (50 %, 70 %, 90 %, and 3 x 100 %) for 10 min each was followed by critical point drying using liquid CO_2. After mounting on aluminium stubs with carbon tabs attached, the specimens were sputter coated with 20 nm gold palladium and viewed using a Hitachi S-4700 scanning electron microscope.

3.3. Inductively Coupled Plasma-Optical Emission spectrometry

The proportion of Pd element in the **Pd-devices** was determined by inductively coupled plasma-optical emission spectrometry at the School of Chemistry of the University of Edinburgh. A small amount of material (1-3 mg) was solubilized in a 10% HNO_3 aqueous solution (1 mL) and heated at 80 °C overnight. The resulting mixture was diluted x5 in distilled water and Pd content measured in a Perkin Elmer Optima 5300 DV ICP-OES spectrometer using commercial Pd sample as standard.

4. Non-biological conversion assays

4.1. Non-biological Pd mediated activation of fluorogenic probe 3

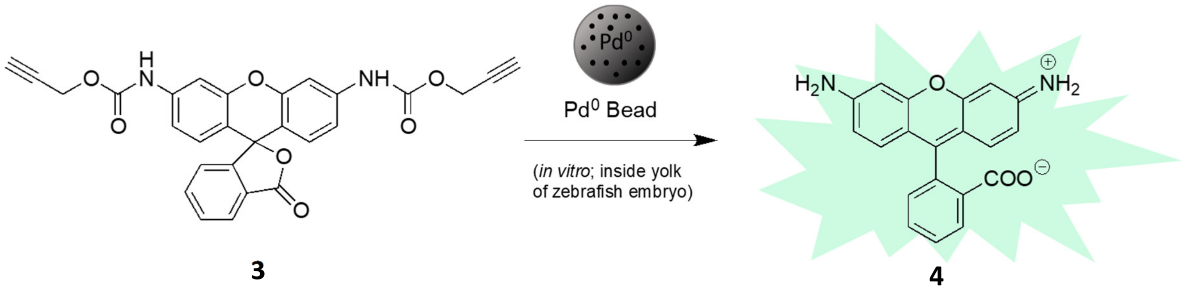

3 4

Probe **3** was synthesised as previously reported [1]. **Pd-devices** (1 mg) were added to a 1 mL solution of reagent **3** (20 μM) in PBS supplemented with 10 % Fetal Bovine Serum (FBS). The mixtures were shaken at 1,200 rpm and 37°C in a thermomixer and reactions monitored at 6 h by fluorescence in a PerkinElmer Victor multilabel reader (excitation filter: 480nm; emissions filter: 535nm). The same conditions and reagents were used to test **Pd-devices** stored at 4 °C for 20 months.

4.2. Non-biological Pd mediated prodrug-into-drug conversion study

Stock sample of **2d** (100 mM in DMSO) was diluted in PBS (1 mL) for a final concentration of 100 μM and incubated with 1 mg of 30 μm **Pd-devices** in a Thermomixer at 37 °C and 1200 rpm. 50 μL aliquots were removed at reported time intervals and monitored by analytical UPLC using a UV detector. Ultra-performance liquid chromatography (UPLC) was performed at Prof Alison Hulme's lab (School of Chemistry, University of Edinburgh) using a Waters Acquity UPLC PDA with a 50 x 2.4 mm Waters BEH C18 1.7 μm column, with compounds detected using an evaporative light scattering detector (ELSD) and UV detector.

5. Biological Methods

5.1. Cell culture

Cell lines were grown in culture media supplemented with 10 % Fetal Bovine Serum (FBS) and 2 mM L-glutamine, then incubated in a tissue culture incubator at 37 °C and 5 % CO_2. Human prostate cancer DU145 cells (a gift from Prof Neil Carragher) and human glioblastoma multiforme U87 cells (a gift from Dr Noor Gamon) were cultured in Dulbecco's modified Eagle's media (DMEM).

5.2. Cytotoxicity study: drug vs prodrug

Cells were seeded in a 96 well plate format at 2000 cells/well and incubated for 48 h before treatment. Each well was then replaced with fresh media containing **1** or **2a-d** and incubated for 5 d. Control cells were incubated with DMSO (0.1 % *v*/*v*). PrestoBlue® cell viability reagent (10 % *v*/*v*) was added to each well and the plate incubated for 1 h. Fluorescence emission was detected using a PerkinElmer Victor2 multilabel reader (Ex / Em: 540 nm / 590 nm). All conditions were normalized to control (100 %) and curves fitted with GraphPad Prism 5 using a sigmoidal variable slope curve.

5.3. Prodrug-into-drug conversion studies

Cells were plated as described and incubated for 48 h prior to treatment. Wells were then replaced with fresh media containing the prodrugs or Pd-devices. For 110 µm Pd-devices studies, appropriate wells were replaced containing 1 mg/mL with DMSO (0.1 % v/v). For 30 µm Pd-devices studies, wells were replaced with fresh media containing 0.6 mg/mL with DMSO (0.1 % *v*/*v*). Wells were replaced with media containing **2a-d** (30 - 3000 nM) with DMSO (0.1 % *v*/*v*); **1** (30 - 3000 nM) with DMSO (0.1 % v/v); or a combination of Pd-devices and prodrugs **2a-d** (30 - 3000 nM) with DMSO (0.1 % v/v). Cell control was incubated with DMSO (0.1 % v/v) alone. All cells were incubated with drugs for 5 d. PrestoBlue® cell viability reagent (10 % *v*/*v*) was added to each well and the plate incubated for 1 h. Fluorescence emission was detected and results normalized as described above.

6. Zebrafish cardiotoxicity study

All zebrafish work presented in this study has been performed in accordance with the UK legal requirements for the protection of animals used for experimental or other scientific research under the European Directive 2010/63/EU and the Animal (Scientific Procedures) Act 1986 amended in 2013. All experiments were performed under the Home Office Project License 70/8000 to EEP. Zebrafish welfare and husbandry were closely monitored by the MRC Human Genetics Unit Zebrafish Facility staff.

Zebrafish (AB line) were raised and maintained as previously described [2]. Embryos were generated by natural pair-wise mating and were kept and handled for all experiments in E3 medium (5 mM NaCl, 0.17 mM KCl, 0.33 mM $CaCl_2$, and 0.33 mM $MgSO_4$). Manually dechorionated embryos were incubated between 24 hpf and 120 hpf[1] in E3 medium containing either **1** (Cambridge Biosciences) or prodrug **2d** at the following concentrations (50 µM, 100 µM, 150 µM and 200 µM). Embryos were soaked in DMSO (0.1 %) and E3 medium as a control. Embryos were kept in 6-well plates (15 embryos / well; 2 wells / condition) at 28.5 °C with dead embryos removed daily. Three experimental replicates were performed with n = 30 (15 x 2) embryos per condition and 2 replicates per condition. Pericardial edema and body surface was estimated using the area measurement tool in Fiji software. Results were obtained by randomly taking > 10 embryos per condition, then determining cardiac/body ratios as previously described [3]. Statistical analyses were performed using Prism GraphPad5 software. Data were subjected to an ordinary one-way ANOVA test. All pictures (fluorescent images included) were taken at the same resolution using an AZ100 upright macroscope (Nikon) with a Plan Apochromat 2 x / 0.45 lens equipped with a Retiga Exi camera (Qimaging) camera. Images were analyzed and processed with Micro-Manager, Fiji and GIMP softwares. Embryos were mounted laterally in 1.5 % Low Melting Point Agarose (Thermo Scientific) and kept in a 0.02 % w/v MS222 (Thermo scientific) solution in E3 medium and culled after imaging in an overdose of MS222 at the end of each experiment.

7. In vivo / ex vivo studies

All murine experiments were approved by the Animal Welfare and Ethical Review Board at the University of Glasgow and performed under the Home Office Project License 30/3185. The human prostate cancer cell line DU145 was purchased from ATCC, authenticated by LCG standards and grown in RPMI (Gibco) containing 10 % (v/v) serum supplement and 2 mM L-glutamine at a temperature of 37 C° with 5 % CO_2. Nude athymic mice were purchased from XimBio. Mice were anesthetized by intraperitoneal injection of 0.5 mg / 1 g body weight NembutalR, 3 µL / 1 g body weight of Ketamine/Xylazine/PBS mixture and subcutaneous morphine was administered subcutaneously at the injection site. The mouse was then placed in an induction chamber with 2 % isoflurane / 98 % oxygen and kept under anaesthesia through continuous nose cone flow during surgery. Mouse was positioned supine, and a midline incision provided access to the prostate gland. A 50 µL cell suspension containing 5 x 10^4 DU145 cells was injected into the prostate, and the round was appropriately closed with metal clips, before the mouse was transferred for recovering and monitoring.

7.1. In vivo intratumoural ultrasound-guided implantation of Pd-devices

Mice identified as having produced tumours from initial cancer cell culture implantation were anaesthetized as above and positioned supine on a mounted platform within a Visual Sonics Vevo 2100 Imaging Station. A solution of 1 mg of 30 µm Pd-devices in 25 µL was suspended in sterile PBS and injected into the mouse prostate using an insulin syringe/needle. Sterile surgical jelly was applied to the injection site, and the needle was appropriately positioned into the plane of ultrasound observation in the prostate tumour. Injection was completed over 30 s and was observed using the enhanced abdominal measurement package in the B-mode setting. Once the needle was removed, mice were transferred for recovery and monitoring.

7.2. Ex vivo catalytic studies

Mice were euthanized according to an IACUC approved method (CO_2 asphyxiation) and an abdominal incision provided access to the prostate. Prostate tumours were excised with scissors, photographed, sliced longitudinally using a rate brain slicer instrument into 1 mm sections and affixed to the bottom of a 24 well plate using 1 - 3 spots of tissue glue at the perimeter of each sample. Tumour slices were soaked in 2 mL of RPMI media containing 10 % (v/v) serum supplement and 2 mM L-glutamine, and 0.1 % (v/v) DMSO dissolving 100 µM of the corresponding reagent.

8. References

[1] Weiss JT et al. *Extracellular palladium-catalysed dealkylation of 5-fluoro-1-propargyl-uracil as a bioorthogonally activated prodrug approach.* Nat. Commun. 2014, 5, 3277.

[2] Kimmel CB, et al. *Stages of embryonic development of the zebrafish.* Developmental Dynamics. 1995; 203(3): 253-310.

[3] Zennaro C, et al. *Podocyte developmental defects caused by adriamycin in zebrafish embryos and larvae: A novel model of glomerular damage.* PLoS One. 2014; 9(5).

[4] Rubio-Ruiz B et al. *Efficient Palladium-Triggered Release of Vorinostat from a Bioorthogonal Precursor.* J. Med. Chem., 2016, 59, 9974–9980.

9. Supplementary Figures

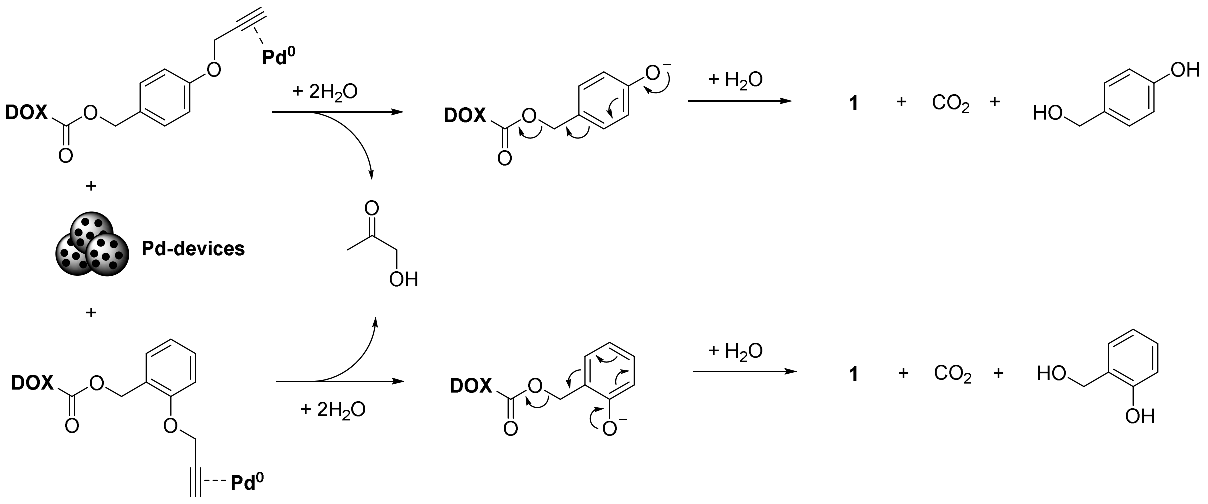

Figure S1. Proposed Pd-triggered uncaging mechanism of PBC-protected doxorubicin prodrugs **2c,d**. The deprotection proceeds via a tandem mechanism triggered by the Pd-catalyzed *O*-depropargylation of a phenolic ether group [4], followed by spontaneous 1,4 or 1,6-elimination of a hydroxybenzyl group with concomitant release of CO_2 and **1**. The quinone methide intermediates are rapidly transformed in non-toxic 1 or 4-hydroxybenzyl alcohol in aqueous media [4].

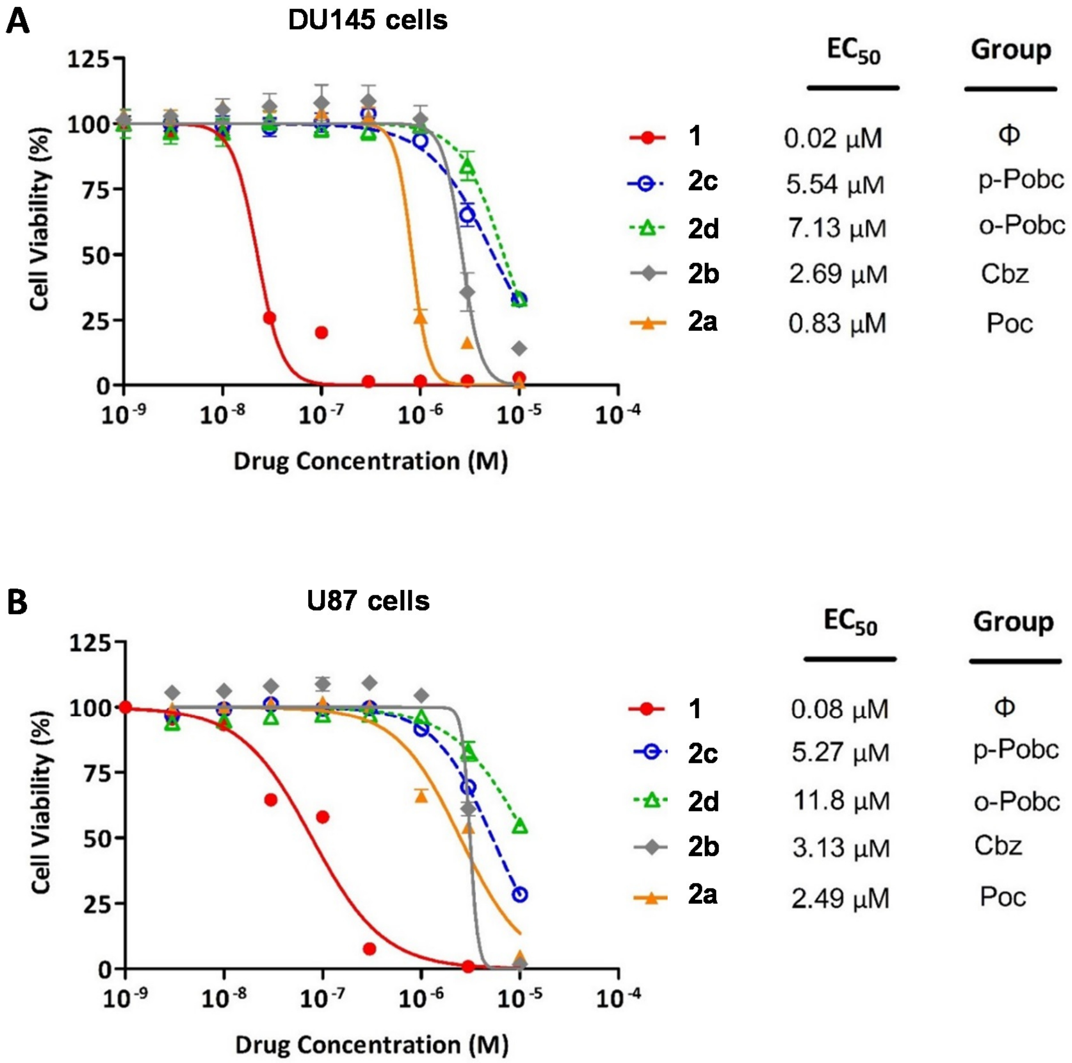

Figure S2. 10-point semilog dose–response curves for **1** and **2a-d** against DU145 and U87 cells.

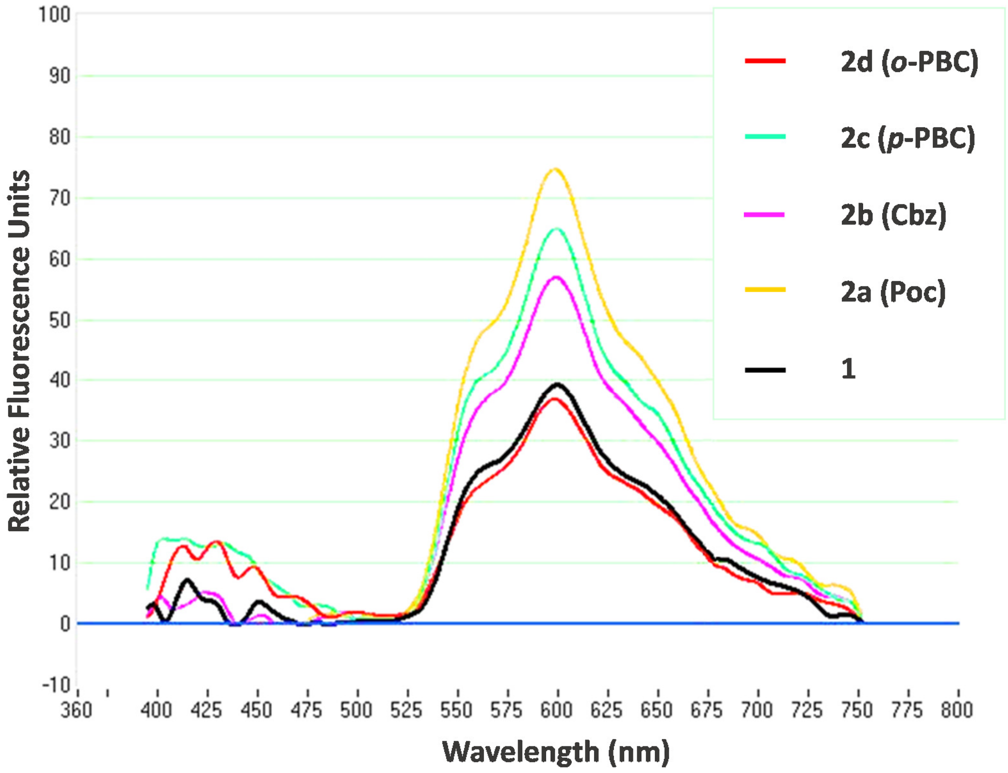

Figure S3. Fluorescence emission spectra of 1 and 2a-b. Protocol: **1** and **2a-d** were incubated at 10 μM in PBS and the fluorescence spectra were collected using a Nanodrop™ 3300 Fluorospectrometer with an excitation wavelength of 365 nm.

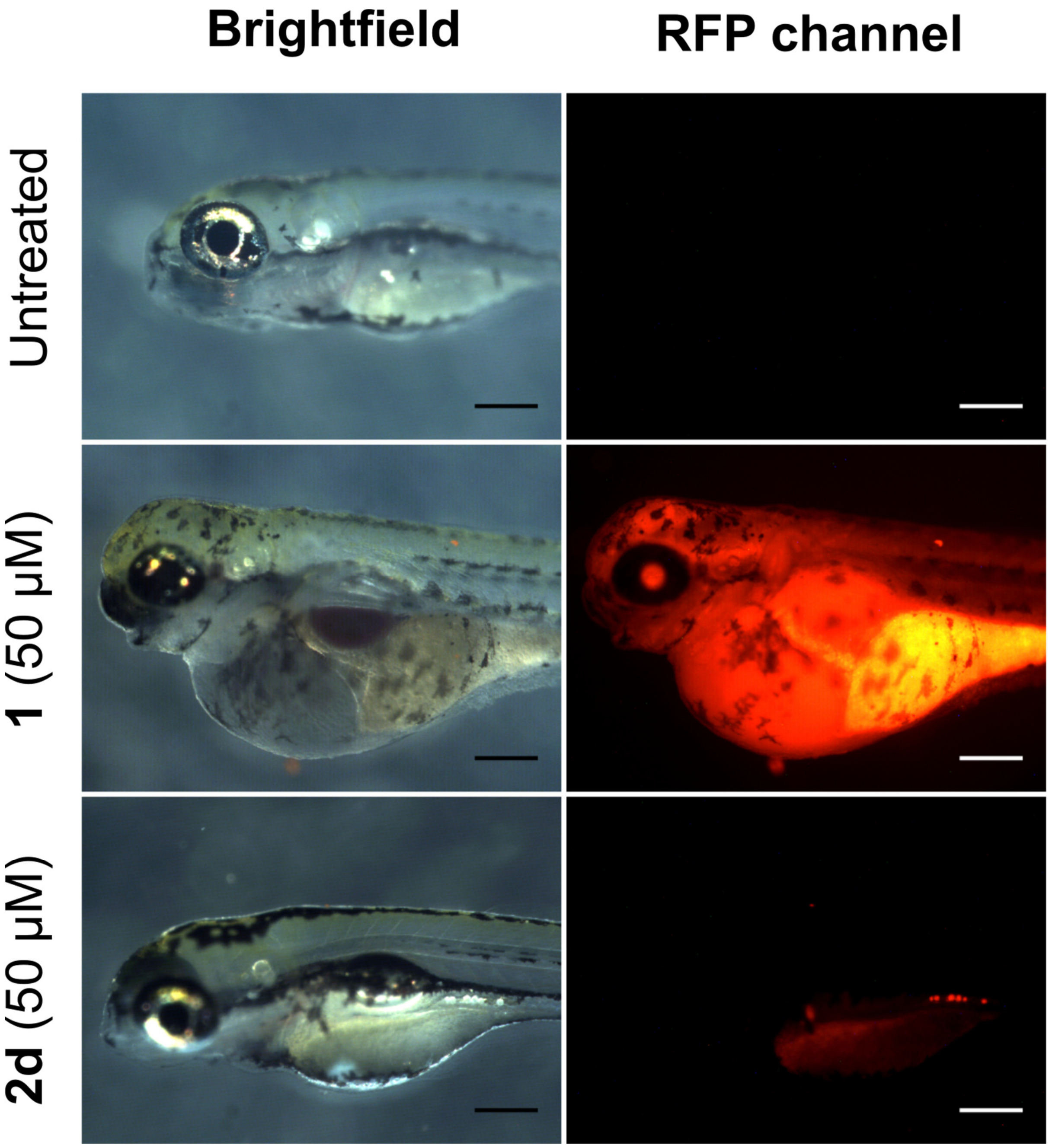

Figure S4. Fluorescence study of 1 and 2d uptake into zebrafish. Lateral-view fluorescence microscopy study of zebrafish embryos treated with **1** and **2d** for 4 d. Larvae were imaged at 120 hpf. Treatments: untreated E3 medium (top), 50 µM of **1** (mid) and 50 µM of **2d** (bottom). Fluorencence images were acquired at same exposure times, resolution and magnification using an AZ100 upright macroscope (Nikon) with a Plan Apochromat 2 x / 0.45 lens equipped with a Retiga Exi camera (Qimaging) camera. Representative images of *n* = 15 fish per condition. Scale bar: 20 mm.

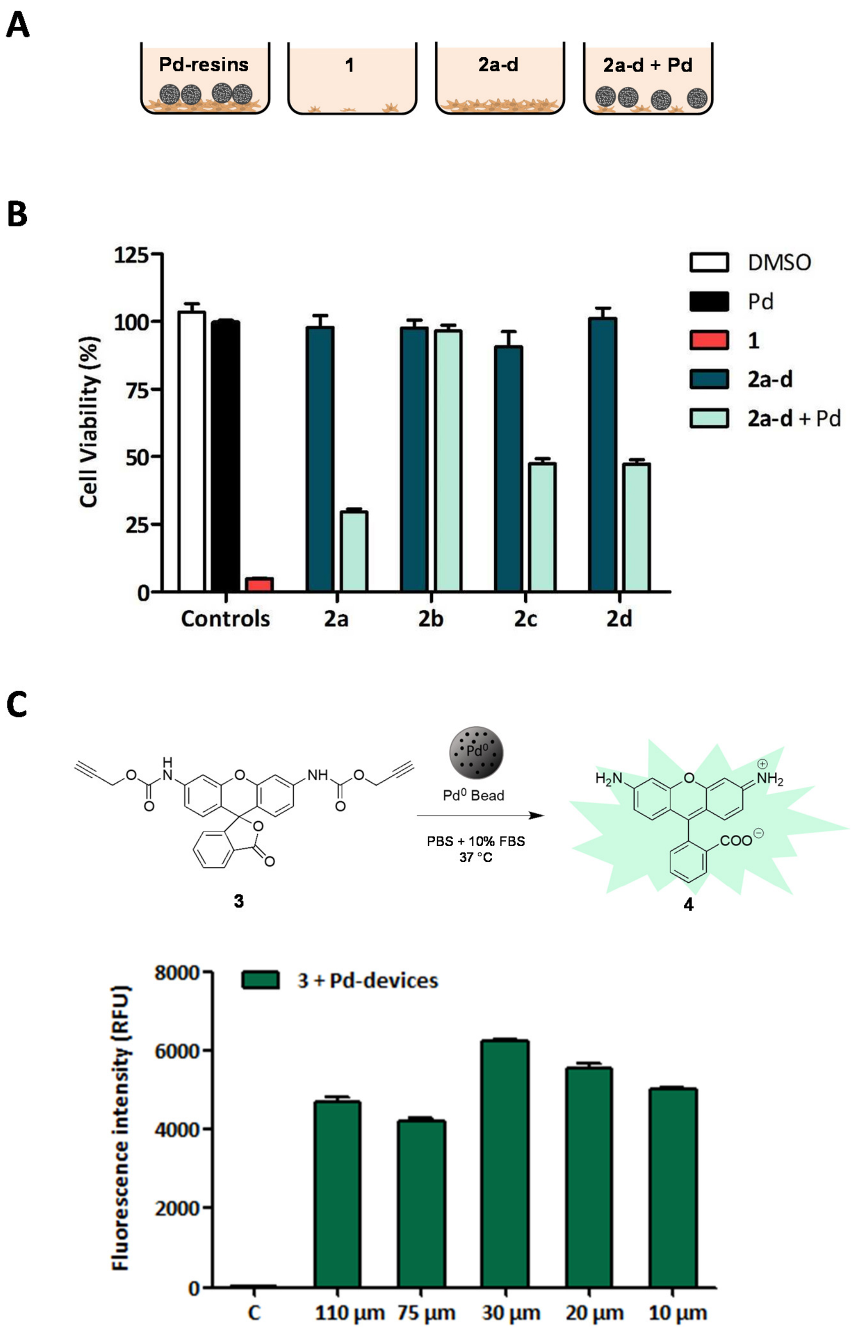

Figure S5. A) Schematic of the prodrug-into-drug conversion study in cell culture. **B)** Pd-mediated activation assay in DU145 cells. Experiments: DMSO only (untreated cell control; white); 1 mg/mL of 110 µm **Pd-devices** (negative control; black); **1** (positive control; red); **2a-d** (negative control; blue); and 1 mg/mL of 110 µm **Pd-devices** + **2a-d** (activation assay; green). All experiments contained 0.1% (*v*/*v*) DMSO. [Drug/prodrug]= 0.3 µM. Cell viability was measured at day 5 using PrestoBlue® reagent. Error bars: ± SD from n = 3. **C)** Pd-mediated fluorogenic assay with **Pd-devices** from 110 to 10 µm in diameter in cell culture conditions. Experiment: prodye **3** (10 µM) was incubated with 1 mg/mL of each **Pd-device** in PBS + 10% FBS in a thermomixer at 37 °C for 6 h at 1200 rpm. Fluorescence intensity was measured at Ex/Em 480/535 nm. Error bars: ± SD from n = 8. Prodye **3** (10 µM) in the absence of Pd was used as negative control.

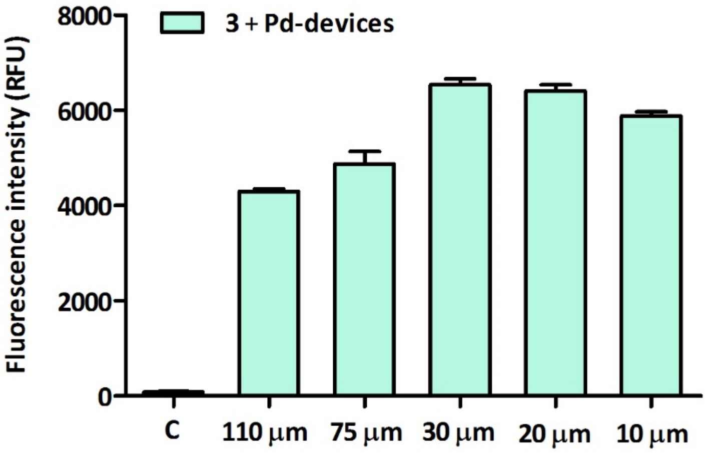

Figure S6. Pd-mediated fluorogenic assay with 20-month old **Pd-devices** (stored at 4 °C) from 110 to 10 μm in diameter in cell culture conditions. Experiment: prodye **3** (10 μM) was incubated with 1 mg/mL of each **Pd-device** in PBS + 10% FBS in a thermomixer at 37 °C for 6 h at 1200 rpm. Fluorescence intensity was measured at Ex/Em 480/535 nm. Error bars: ± SD from n = 8. Prodye **3** (10 μM) in the absence of Pd was used as negative control.

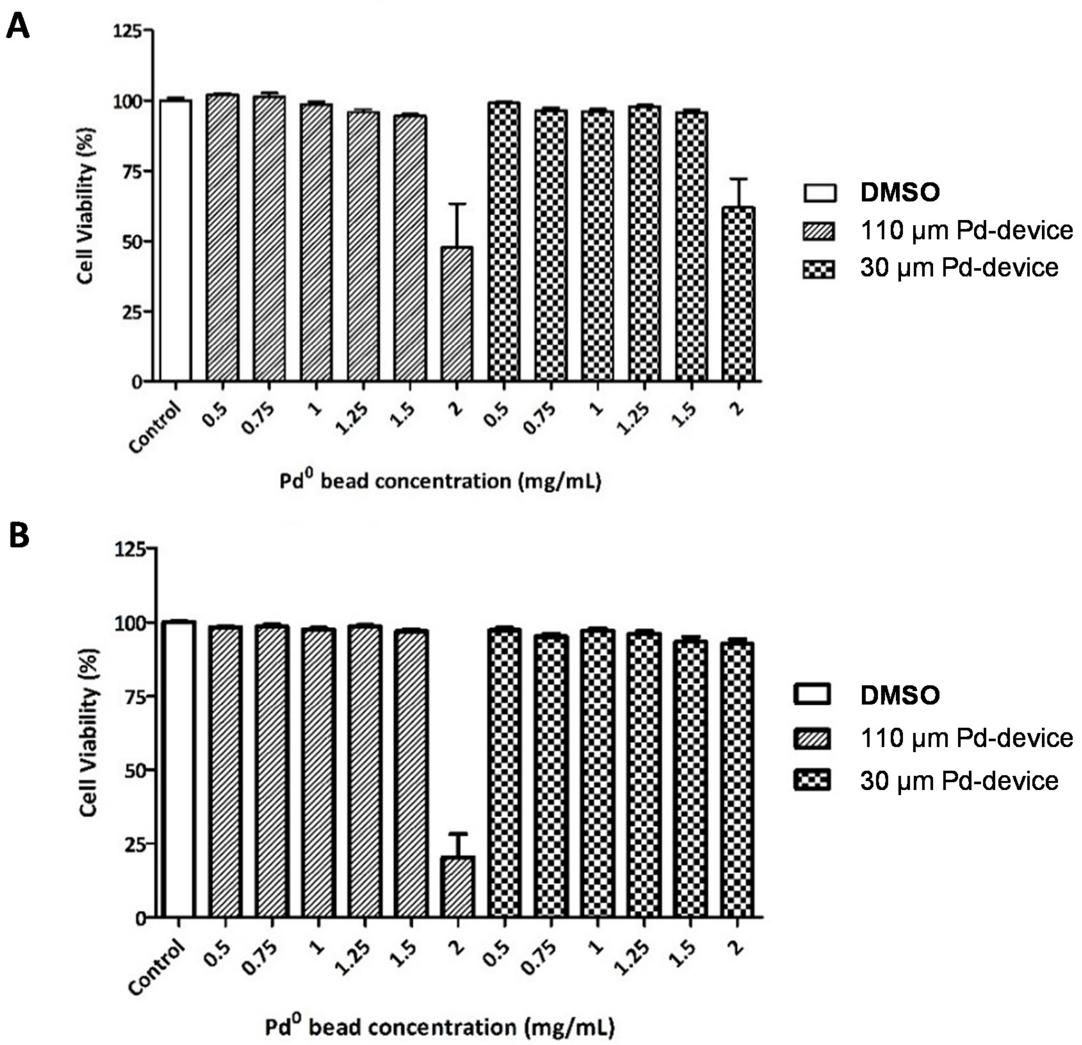

Figure S7. Biocompatibility of 30 µm vs 110 µm Pd-devices in cell culture. Study of the unspecific cytotoxic effect induced by incubating increasing concentrations (0.5 – 2 mg / mL) of 30 µm **Pd-devices** (hatched) and 130 µm **Pd-devices** (checked) in (**a**) DU145 and (**b**) U87 cell culture. Cell viability was measured at day 5 using PrestoBlue® reagent. Error bars: ± SD from *n* = 6.

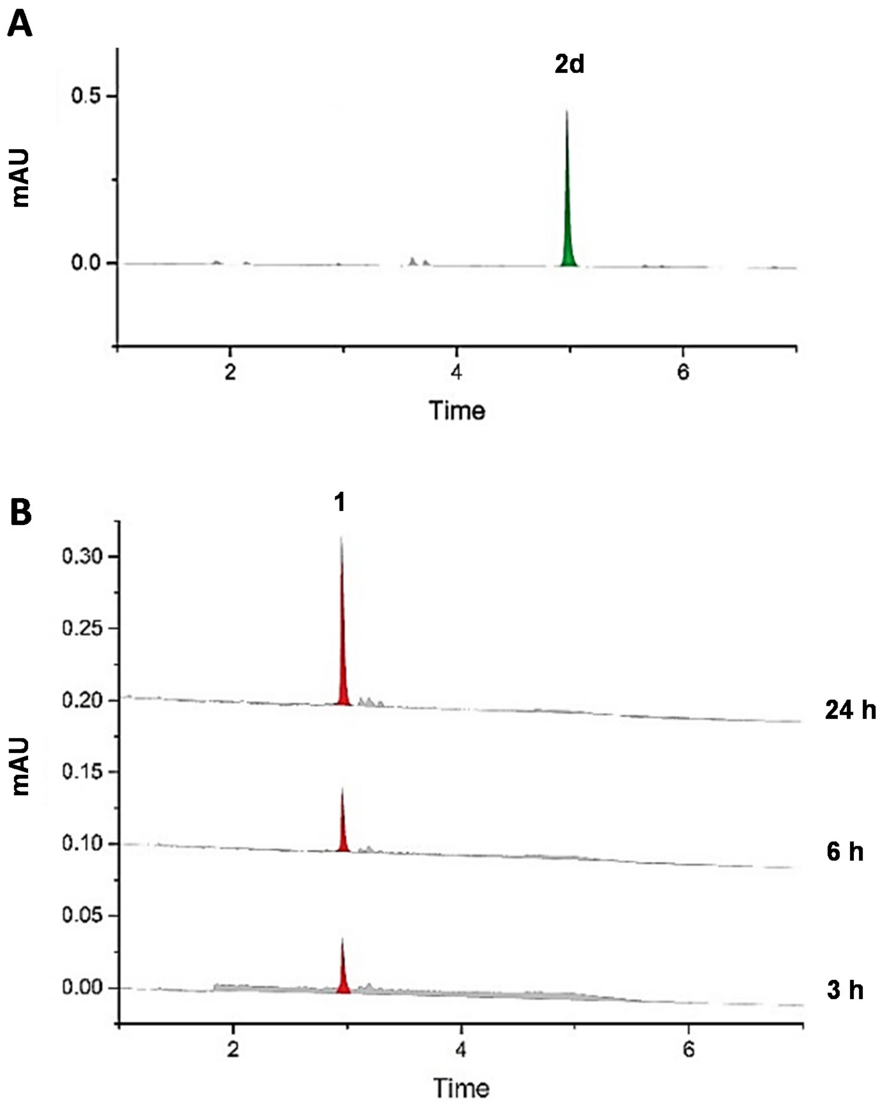

Figure S8. Non-biological prodrug-into-drug conversion study with 30 µm Pd-devices. A) UPLC chromatogram of prodrug **2d**. **B)** UPLC chromatogram of the reaction of **2d** (100 µM in PBS) with 1 mg / mL of 30 µm **Pd-devices** incubated in a Thermomixer at 37 °C and 1400 rpm for 3, 6 and 24 h.

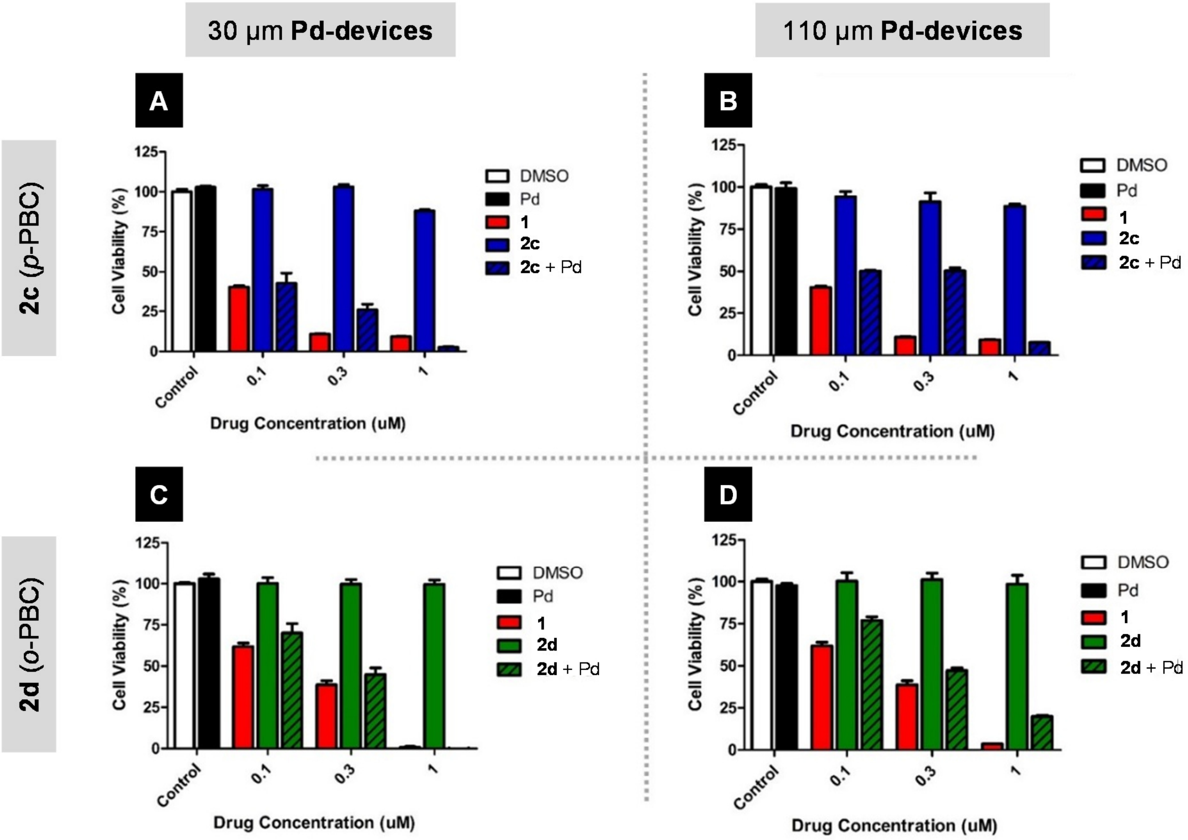

Figure S9. Comparative Pd-mediated activation of 2c and 2d with 30 µm vs 110 µm Pd-devices in DU145 cell culture. Toxigenic effect of **2c,d** in the presence of **Pd-devices** (1 mg/mL) was determined at three different concentrations: 0.1, 0.3 and 1 µM. Experiments: 0.1 % (v/v) DMSO (untreated cell control, white); 1 mg/mL of (**A,C**) 30 µm or (**B,D**) 110 µm Pd-devices (negative control, black); **1** (drug / positive control, red); prodrug **2c** (blue) or **2d** (green); combination of **2c,d** (striped) + 30 µm or 110 µm **Pd-devices** (striped bars). Cell viability was measured at day 5 using PrestoBlue® reagent. Error bars: ± SD from *n* = 3.

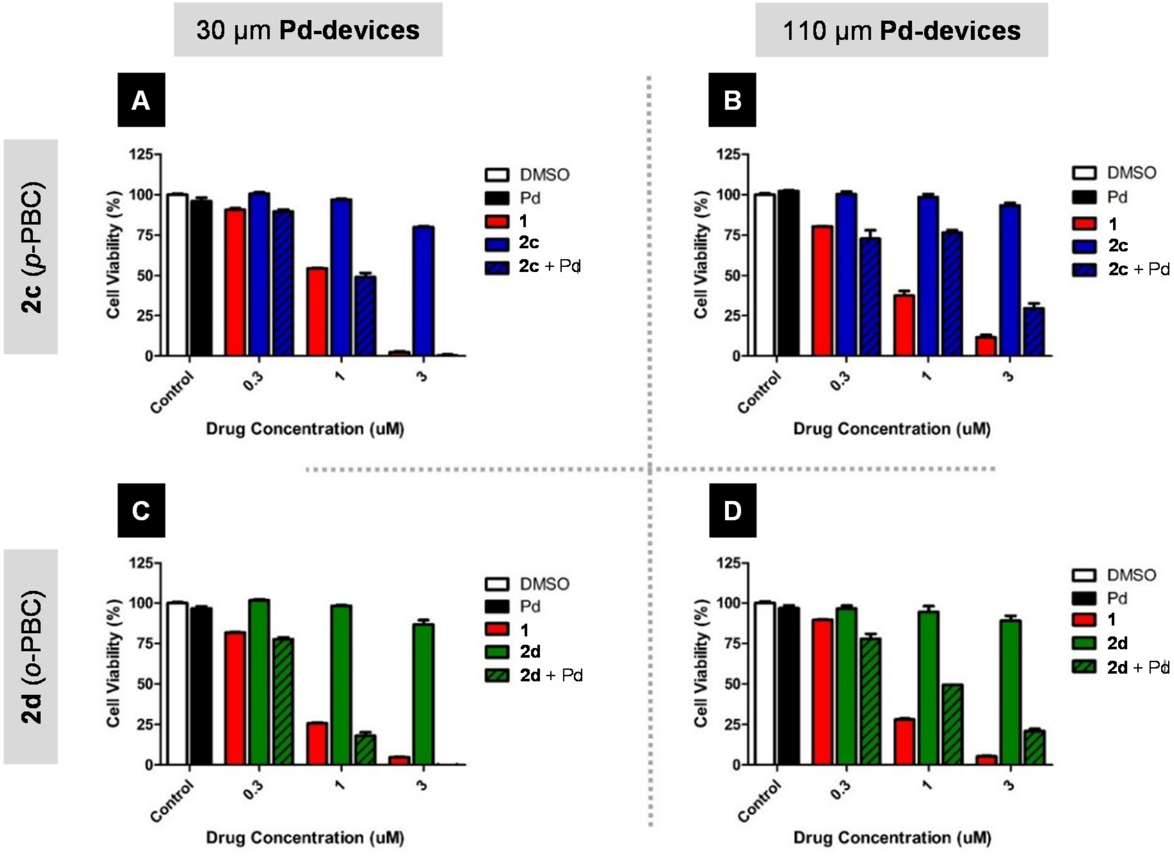

Figure S10. Comparative Pd-mediated activation of 2c and 2d with 30 µm vs 110 µm Pd-devices in U87 cell culture. Toxigenic effect of **2c,d** in the presence of **Pd-devices** (1 mg/mL) was determined at three different concentrations: 0.1, 0.3 and 1 µM. Experiments: 0.1 % (v/v) DMSO (untreated cell control, white); 1 mg/mL of (**A,C**) 30 µm or (**B,D**) 110 µm Pd-devices (negative control, black); **1** (drug / positive control, red); prodrug **2c** (blue) or **2d** (green); combination of **2c,d** (striped) + 30 µm or 110 µm **Pd-devices** (striped bars). Cell viability was measured at day 5 using PrestoBlue® reagent. Error bars: ± SD from *n* = 3.

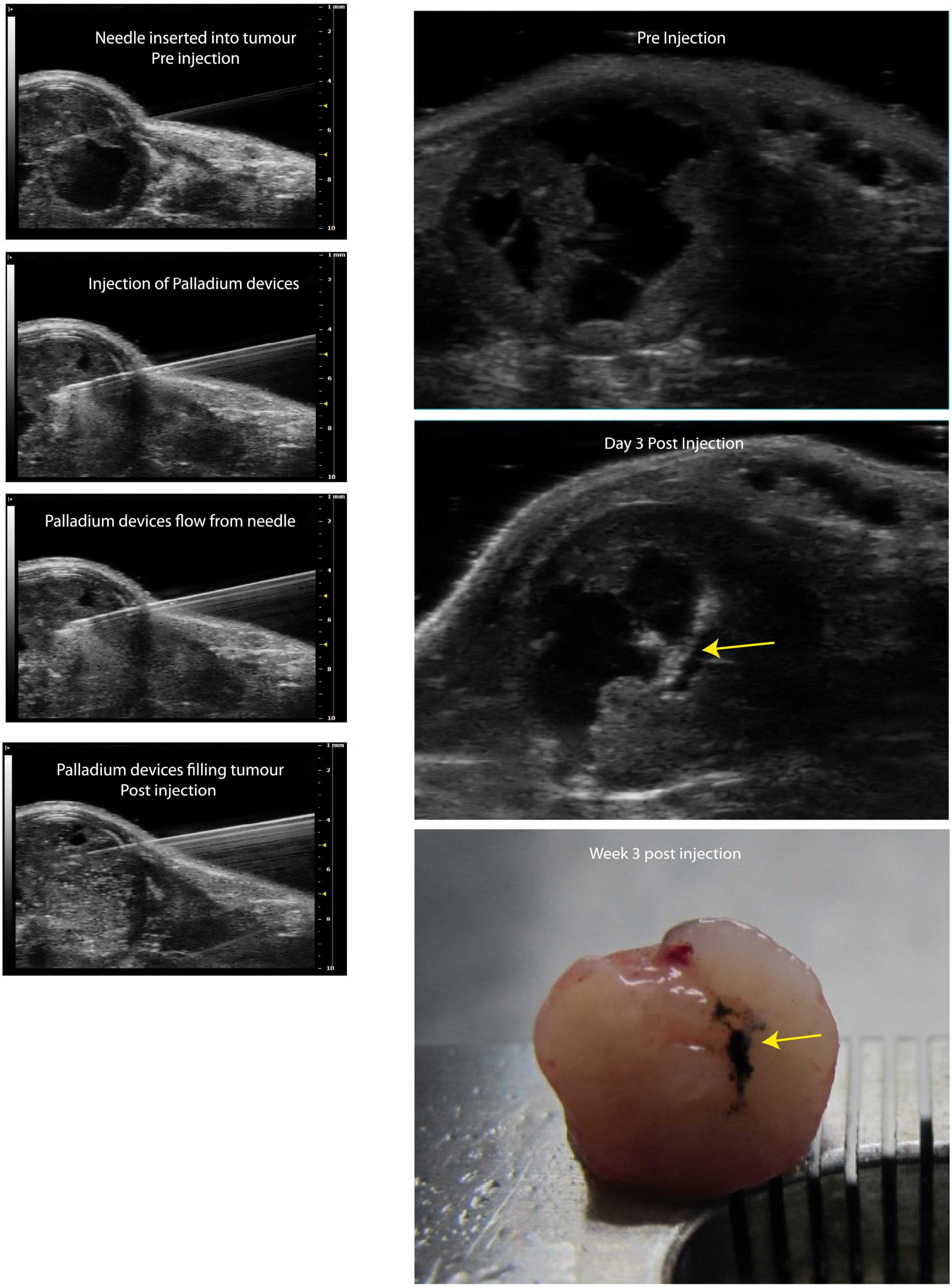

Figure S11. In vivo ultrasound imaging analysis pre and post-injection of Pd-devices. After mice sacrifice at day 21, tumours were resected and halved to visualise intratumoural Pd-implant distribution (bottom photo).

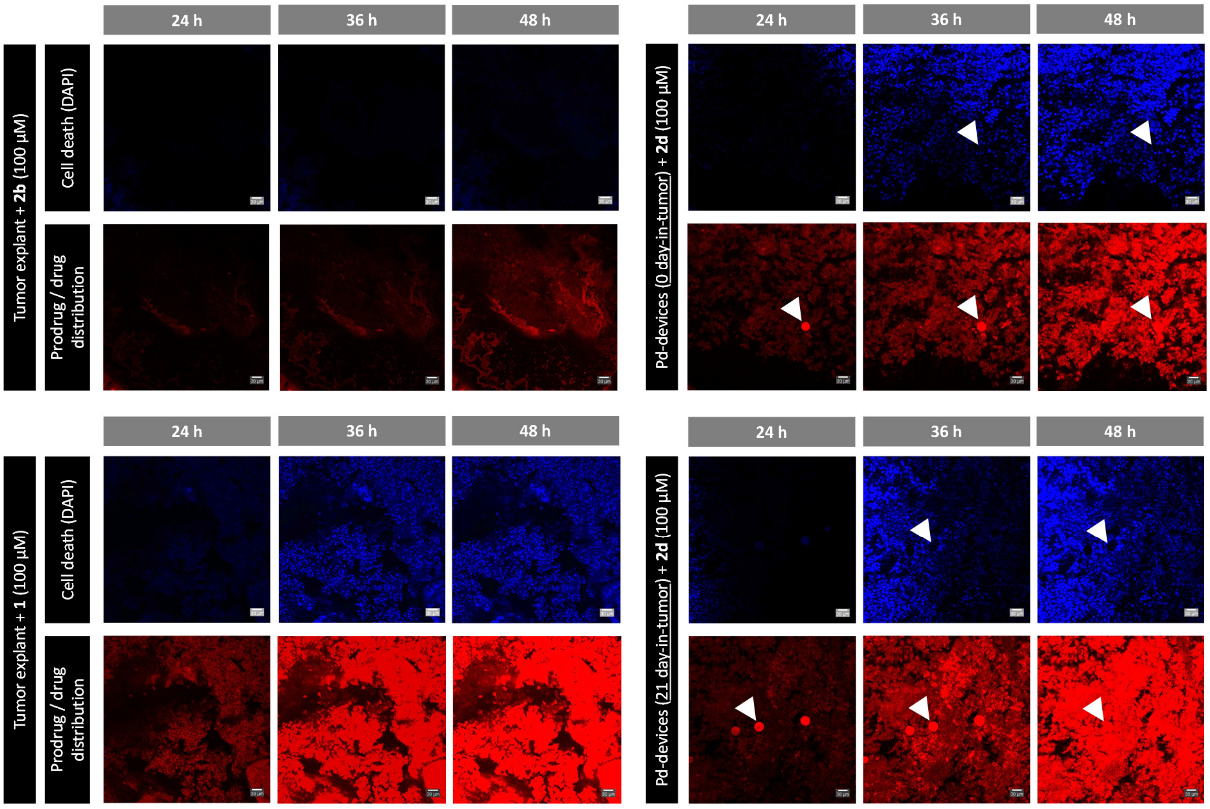

Fig S12. Live-cell imaging study of in human DU145 prostate tumour explant model. (Left) Images of a representative tumour explant (n = 2) after treatment with **2d** (100 µM, top panel) or **1** (100 µM, bottom panel). Pictures taken at 24, 36 and 48 h. **(Right)** Pd-mediated conversion of inactive **2d** into cytotoxic **1**. Images of a representative tumour explant (n = 2) bearing **0**- or **21-day-in-tumour Pd-devices** after treatment with **2d** (100 µM) and DAPI. Images taken at 24, 36 and 48 h. Tissue samples were imaged under 460 nm (for DAPI staining, in blue) and 560 nm (for **2d**/**1** distribution, in red) laser excitation. The presence of the **Pd-devices**, indicated with white arrows, is identified by a bright fluorescence signal in the red channel. Pictures were generated using ImageJ software. Scale bar = 30 µm.

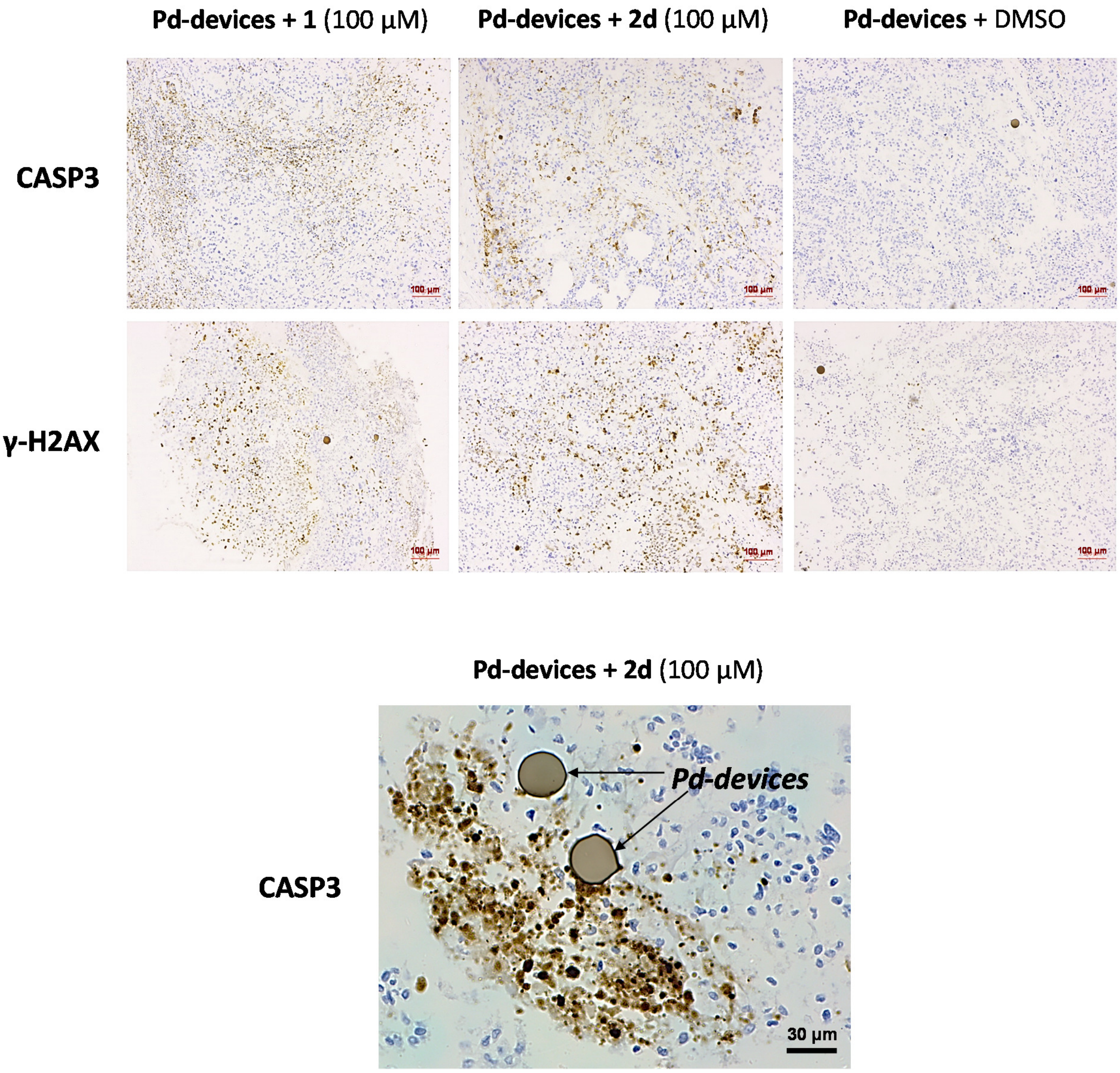

Figure S13. Immunohistochemistry analysis of a tumour explant containing 21-day-in-tumour Pd-devices after treatment with either 1, 2d or DMSO. Caspase 3 (apoptotic biomarker) or γ-H2AX (double-strand DNA breaks) antibody staining of DU145 prostate tumour explants containing **Pd-devices**. Antibody staining is identified as brown color. Note that **Pd-devices** are identified as brown-greyish circles of 30 µm of diameter.
